# Structural insights into the outer membrane proteins PorA, OMP50 and Cj0034c from native *Campylobacter jejuni* membranes

**DOI:** 10.1371/journal.pbio.3003961

**Published:** 2026-08-28

**Authors:** Zhemin Zhang, William D. Gregor, Muslum Ilgu, Yue Yin, Philip A. Klenotic, Qijing Zhang, Edward W. Yu

**Affiliations:** 1 Department of Pharmacology, Case Western Reserve University School of Medicine, Cleveland, Ohio, United States of America; 2 Department of Veterinary Microbiology, College of Veterinary Medicine, Iowa State University, Ames, Iowa, United States of America; 3 Department of Molecular Biology and Microbiology, Case Western Reserve University School of Medicine, Cleveland, Ohio, United States of America; Rutgers University-Robert Wood Johnson Medical School, UNITED STATES OF AMERICA

## Abstract

Bacterial outer membrane proteins (OMPs) are critical players in host–pathogen interactions and environmental adaptation. Here we describe the newly developed “Gradient Enrichment of Native Targets from Lipid Environments” (GENTLE) methodology and use this approach to elucidate the structures of *Campylobacter jejuni* OMPs directly from native, detergent-solubilized crude membranes. We identify and solve high-resolution cryo-EM structures of PorA, OMP50, and Cj0034c from *C. jejuni* membranes, all of which are required for *Campylobacter* invasion, adhesion, and initiation of host infection. Notably, our results provide the first structural information of OMP50, revealing a two-domain architecture constructed with an all β-stranded transmembrane domain and an all α-helical periplasmic domain. This structure depicts that all tyrosine residues, many of which are expected to be critical for phosphorylation and host–pathogen interaction, are localized to the outer membrane of *C. jejuni*. Our studies also led to the first structure of the full-length Cj0034c protein, which assembles as a nonamer with each protomer containing a single-spanning transmembrane helix and a large periplasmic domain. The nine protomers stack side-by-side to form a channel that spans the entire lipid bilayer. However, whether Cj0034c spans the outer membrane (OM) or inner membrane (IM) of *C. jejuni* must await further experimental studies. In addition, we observed that the surface-exposed extracellular loop L4 of PorA is very flexible, which may be critical for the virulence of this porin. Collectively, this work provides novel structural information for functionally important OMPs and sheds light on how they assemble in native bacterial membranes. These findings further demonstrate that it is possible to obtain high-resolution structural information for targeted membrane proteins from crude native membranes without their overexpression and purification.

## Abstract 2

Bacterial outer membrane proteins mediate host-pathogens interactions, but many aspects remain unclear. This study develops a GENTLE high-resolution cryo-EM method that reveals new architectures and assembly mechanisms for the Campylobacter jejuni proteins PorA, OMP50, and Cj0034c.

## Introduction

Membrane proteins are major components of both prokaryotes and eukaryotes, constituting 20% to 30% of the proteome [1,2]. They play critical roles in cellular functions such as intercellular communication, transmembrane signaling, and transport of molecules in and out of the cell. These membrane proteins can be classified into two distinct categories: α-helical and β-barrel transmembrane proteins [2]. The α-helical transmembrane proteins are commonly found in plasma membranes of eukaryotic cells and inner membranes (IMs)/cytoplasmic membranes of bacterial cells, whereas the β-barrel transmembrane proteins are exclusively observed in outer membranes (OMs) of mitochondria, chloroplasts, and gram-negative bacteria [3]. In contrast to α-helical IM proteins (IMPs), which constitute the majority of bacterial membrane proteins, β-barrel OM proteins (OMPs) comprise less than 3% of proteins encoded in bacterial genomes [4]. In bacterial pathogens, membrane proteins are essential for environmental adaptation and host–pathogen interactions. Therefore, their structural information is instrumental for the understanding of cellular communication, signaling, membrane transport, host–pathogen interaction, and the progress of drug design and development. However, the process of solving high-resolution structures of these membrane proteins often requires tedious and complicated overexpression and purification procedures. As a result, the structural information obtained after these procedures, particularly the stoichiometry, oligomerization, and assembly of membrane protein complexes, has often been found to be controversial, thus pressuring the development of structural biology techniques capable of elucidating target proteins and protein complexes in native membranes.

In this study, we aim to overcome these limitations to obtain high-resolution structural information of *Campylobacter jejuni* OMPs from crude native membranes without protein overexpression and purification. *C. jejuni* is a leading foodborne pathogen, causing gastroenteritis and accounts for > 400 million cases of diarrhea each year worldwide [5,6]. Enteritis is the primary clinical indicator of campylobacteriosis [7]. Symptoms of the infection include hematochezia, diarrhea, cramping, abdominal pain, fever, nausea, and vomiting. In addition, extraintestinal infections such as bacteremia [8,9], hepatitis [10] and pancreatitis [11] may occur. Unfortunately, some patients develop post-acute infection syndromes including reactive arthritis [12], and neurological disorders such as Guillain–Barré syndrome [6], to further complicate the treatment. *C. jejuni* also plays a significant role in more than 20% of deaths under the age of 5, where diarrhea is the major cause of morbidity and mortality [13]. Owing to the rising prevalence of antibiotic-resistant *Campylobacter*, the Centers for Disease Control and Prevention (CDC) have classified antibiotic-resistant *Campylobacter* as a serious antibiotic resistance threat in the United States [14,15].

In gram-negative bacteria, the OM of the cell envelope is a very critical layer that creates the primary interface with the host. The OM has the ability to regulate membrane permeability in response to different media, pH and temperature, as well as host immunity [16–18]. The pore-forming OMPs, termed porins, govern membrane permeability and exchanges between bacteria and their environment. They are the essential apparatus that allows soluble substances and hydrophilic molecules to pass in and out of the OM. In *E. coli*, OmpF and OmpC are the most prevalent OM porins [19] and serve as models for other gram-negative porin proteins in relation to their regulation, expression and function.

In *C. jejuni*, OMPs are considered to be of particular importance in order to establish a successful infection in the host. To colonize the mucosal surface, bacterial microorganisms require adherence factors such as pili, which are surface structural elements expressed by many bacteria to facilitate the anchoring of bacterial cells to the host [20]. However, genome annotations of strains of *C. jejuni* do not reveal obvious pilus-like genes, suggesting that *C. jejuni* may utilize distinct mechanisms to host cells [21]. It has been observed that colonization of the host intestine by *C. jejuni* is promoted by surface flagellum-mediated motility, such as Cia, Fed and Fla, and fibronectin-mediated binding, such as Cad and Flp, to the host [22–24]. In addition, several surface-exposed OM porins are implicitly implicated in colonization, where two porins, major outer membrane protein (MOMP) or porous protein A (PorA) [25–28], and outer membrane protein 50 (OMP50) or *C. jejuni* tyrosine kinase (CjTK) [29,30], have been identified and characterized. PorA forms a relatively large channel that allows larger-sized solutes to pass through the OM. It has the highest expression level among all porins residing at the OM of *C. jejuni* [31]. OMP50 is considered a minor OM porin that forms a relatively small channel, presumably decreasing membrane permeability and only allowing smaller molecules to pass through the OM [29,32]. In addition to serving as porins, PorA plays an important role in systemic spread of *C. jejuni* [27], while OMP50, functioning as a tyrosine kinase, controls the phosphotyrosine network and regulates capsule production in *Campylobacter* [30]. Despite the importance of these OMPs in *Campylobacter* pathophysiology, their native structural conformations and assembly in the membrane remain poorly understood. This has hindered the ability to acquire an in-depth understanding of *Campylobacter* pathogenesis and ensuing development of mitigation strategies.

We recently developed a cryo-EM methodology termed “Build-and-Retrieve” (BaR) to simultaneously identify and solve high-resolution structures of proteins, enzymes, and their complexes from crude biological samples [33]. We also employed this methodology to create a platform to elucidate overexpressed membrane protein complexes from native membranes, and this allowed us to solve cryo-EM structures of the *Mycobacterium smegmatis* MmpL5-MmpS5 complex system from crude *M. smegmatis* membranes [34]. To expand upon our efforts to elucidate high-resolution structural information of membrane proteins from their respective native membranes without overexpression and purification, we developed a methodology, termed “Gradient Enrichment of Native Targets from Lipid Environments” (GENTLE) (Fig 1). This method couples sucrose cushion ultracentrifugation with the BaR cryo-EM technique to enrich the population of protein particles. The idea behind this approach is to enhance the chance of identifying and elucidating structures of membrane proteins and complexes in native membranes. We hypothesized that this methodology can be applied to facilitate structural determination of porins critical for virulence, pathogenesis, and host–pathogen interaction residing at the *C. jejuni* OM.

**Fig 1:**
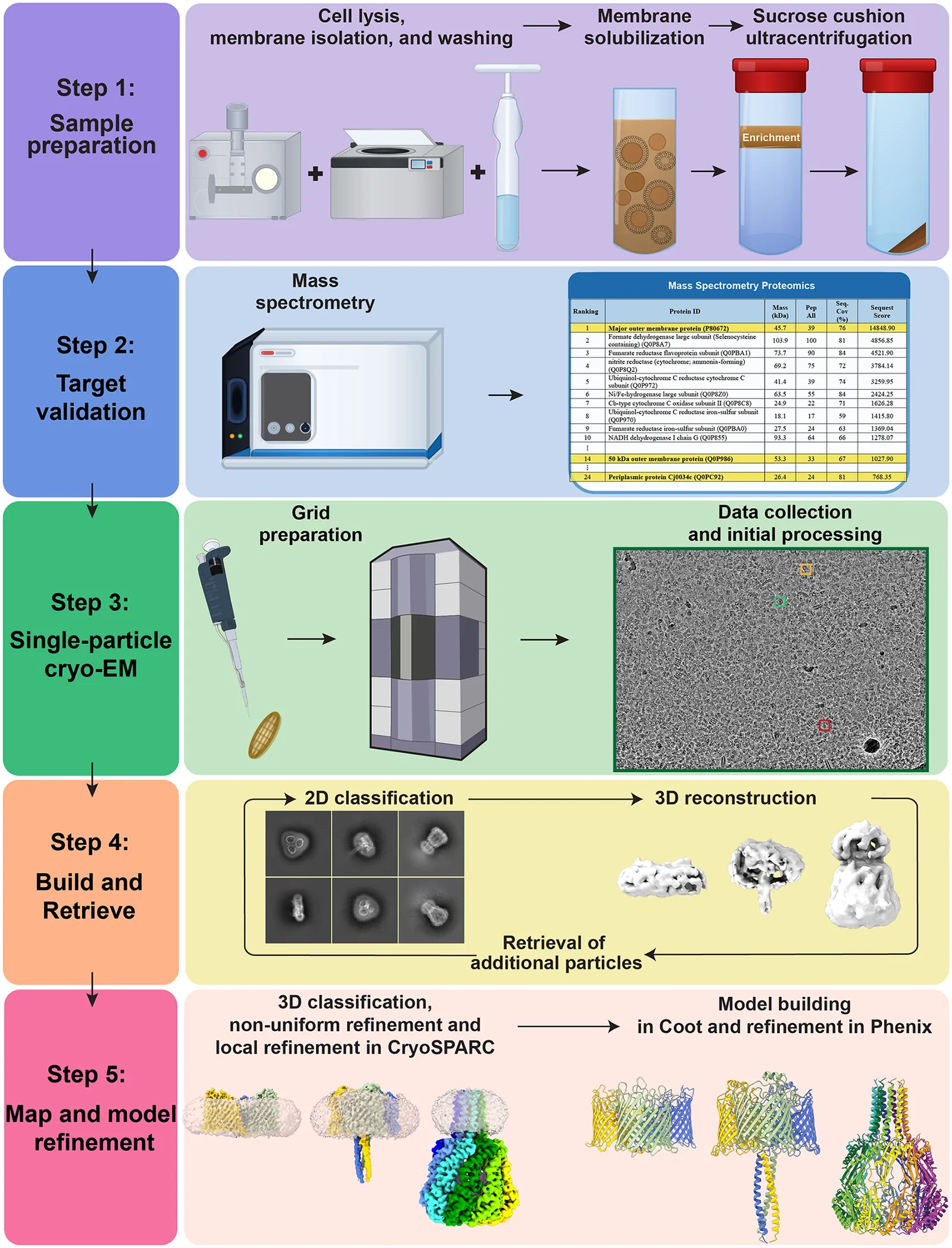
Overview of the GENTLE methodology. Details of GENTLE can be found in the step-by-step GENTLE protocol and experimental procedures included in the Methods section.

Here we use the GENTLE methodology to simultaneously identify and solve high-resolution structures of three different membrane proteins from crude, detergent-solubilized native *C. jejuni* membranes. From this sample, we identified and solved structures of PorA/MOMP, OMP50/CjTK, and Cj0034c, also known as CjSLP (*C. jejuni* signaling molecule that interacts with mouse pelle-like kinase (Simp)-like protein). Our work provides the first structural information of OMP50 and Cj0034c. It also leads to the observation that the flexibility of the surface-exposed extracellular loop L4 of PorA may be critical for the virulence of this porin.

## Results

### “Gradient Enrichment of Native Targets from Lipid Environments” (GENTLE) methodology

Application of the GENTLE methodology allowed us to identify and solve cryo-EM structures of OMPs from native *C. jejuni* membranes without overexpression and purification of our protein targets. We grew *C. jejuni* NCTC 11168 cells, harvested their membranes, solubilized them in detergent micelles, and enriched the membrane protein components using sucrose cushion centrifugation. Mass spectrometry confirmed the presence of OMPs in this *C. jejuni* membrane sample (S1 Table). This enriched sample was then applied to a holey carbon grid and single-particle cryo-EM images were collected. The ensuing cryo-EM data were processed using the BaR methodology [33], where *in silico* purification and sorting of images were able to isolate and separate the images into different classes. 2D classifications of the images indicated that there are at least two distinct classes of images resembling the architecture of OMPs. In addition, a third class of images depicting a membrane protein that likely possess a large periplasmic domain was detected. Several iterative rounds of 2D/3D classifications allowed us to rigorously sort these images (S1 Fig). Based upon these classes of single particles, we were able to identify and solve high-resolution cryo-EM structures of the PorA/MOMP and OMP50/CjTK OM porins. Additionally, we obtained a high-resolution cryo-EM map of the protein images subsequently identified as the virulence factor Cj0034c/CjSLP (S1 Fig and S2 Table). The Step-by-step GENTLE protocol is listed as follows:

***Step 1: Sample preparation.*** The preparation procedures entail cell growth, cell lysis, membrane isolation, solubilization of membranes in detergent (such as lauryl maltose neopentyl glycol (LMNG) or glycol-diosgenin (GDN)), and the enrichment of protein components of solubilized membranes using sucrose cushion ultracentrifugation.

***Step 2: Target validation.*** The presence of target proteins is confirmed using mass spectrometry and proteomic analysis.

***Step 3: Single-particle cryo-EM.*** Membrane sample is loaded onto cryo-EM grids followed by cryo-EM data collection, initial image processing, and preliminary 2D and 3D ab initio classifications.

***Step 4: Build and Retrieve.*** Different subsets of 3D classes are vigorously cleaned and re-sorted via several rounds of 2D classifications and ab initio 3D reconstructions to build initial maps. The built maps are then used as templates for 3D heterogeneous classifications of the 2D cleaned particles, where each subset is treated separately to retrieve additional particles for higher resolution reconstruction.

***Step 5: Map and model refinement.*** Retrieved subsets are cleaned via multiple rounds of 2D and 3D classifications, and non-uniform refinements performed to build the final maps. Symmetry can be applied to improve the resolutions of the maps. As soon as the resolutions of the cryo-EM maps reach ~3.5 Å or better, these maps are used to determine their corresponding identities using the program DeepTracer [35]. Models are then constructed using Coot [36] and refined using PHENIX [37].

Detailed procedures, including sample preparation, proteomic analysis, cryo-EM data collection and processing, building maps and retrieving single particles, and map and model refinements are described in the Methods section.

### PorA/MOMP

The most abundant OM porin that we identified is the PorA membrane protein (S1 Fig). PorA was named MOMP because it is highly abundant at the OM of *C. jejuni*. It creates a relatively large pore on the cell surface to allow for the exchange of solutes and solvents in and out of the *C. jejuni* cell [26,38]. PorA appears to be a moonlighting protein that performs multiple functions. It has been reported that PorA functions not only as a porin, but also as an adhesin to anchor the bacterium at the host’s intestinal epithelium [25,38,39]. The myriad of genetic polymorphisms of the *porA* gene (*cj1259*), particularly in surface-exposed regions of expressed PorA variants, often allow the bacterium to bypass the host’s immune response [40]. The associated virulence of this porin has been highlighted by the discovery of the antibiotic-resistant and hypervirulent strain, named clone “sheep abortion” (SA) [41,42], where mutations within one of the cell surface-exposed loops of PorA are responsible for the hypervirulence [27].

***Structure of PorA-I.*** We collected a total of 136,178 single-particle projections for this class of images. Rigorous 2D/3D classifications allowed us to sort these projections into two subclasses with similar structural features. The first subclass contains 85,621 single particles with the generated cryo-EM map at 2.47 Å resolution. The program DeepTracer [35] led us to confirm the identity of this membrane protein as full-length PorA. We then solved its cryo-EM structure, designated as PorA-I, at this resolution (S2 Table). The structure of PorA-I resolved from crude *C. jejuni* membranes depicts that this porin assembles as a trimer. Each protomer forms an 18-stranded β-barrel (β1-β18) protein, where this barrel creates a pore to allow for substrate transport (Figs 2A–2C and S2). Therefore, PorA possesses three pores within the trimer. This structural assembly is in good agreement with the X-ray structure of this protein [26]. Superimposition of our cryo-EM structure to the X-ray structure of PorA (PDB ID: 5LDT) [26] gives rise to a root-mean-square-deviation (r.m.s.d.) of 0.6 Å (S3 Fig). Each PorA protomer also contains nine extracellular loops (L1–L9) and eight periplasmic loops (T1–T8), connecting the 18 β-strands which form the core of the β-barrel (Figs 2C and S2). Interestingly, L3, L4 and L7 fold into the middle of the pore formed by the 18-stranded β-barrel (Fig 2C). These loops also create the narrowest region of each pore (Fig 2D). Residues 171–177 of L4 create an α-helix (α2) facing toward the outer leaflet of the OM. An extra spherical-shaped cryo-EM density surrounded by L3, L4 and L7 was found in the middle of each PorA protomer. The location of this extra density overlaps with a Ca^2+^ ion found in the X-ray structure of PorA [26]. We therefore assigned this extra density as a Ca^2+^ ion, which forms bonds with D142 of L3, Q174 and D177 of L4, and E307 of L7 (Fig 2C). These negatively charged and polar residues along with helix α2 appear to be critical for ion binding. Electrostatic surface potential calculations indicate that the extracellular surface of the PorA β-barrel is predominantly neutral, but residues at locations corresponding to the outer leaflet of the OM make up positively and negatively charged patches (Fig 2E). Interestingly, the pore formed by each PorA protomer is negatively charged in nature (Fig 2E), suggesting that PorA may favor the shuttling of positively charged substrates. This result is indeed in good agreement with the observation that PorA is capable of transporting small positively charged poly-arginine peptides across the membrane [43].

**Fig 2:**
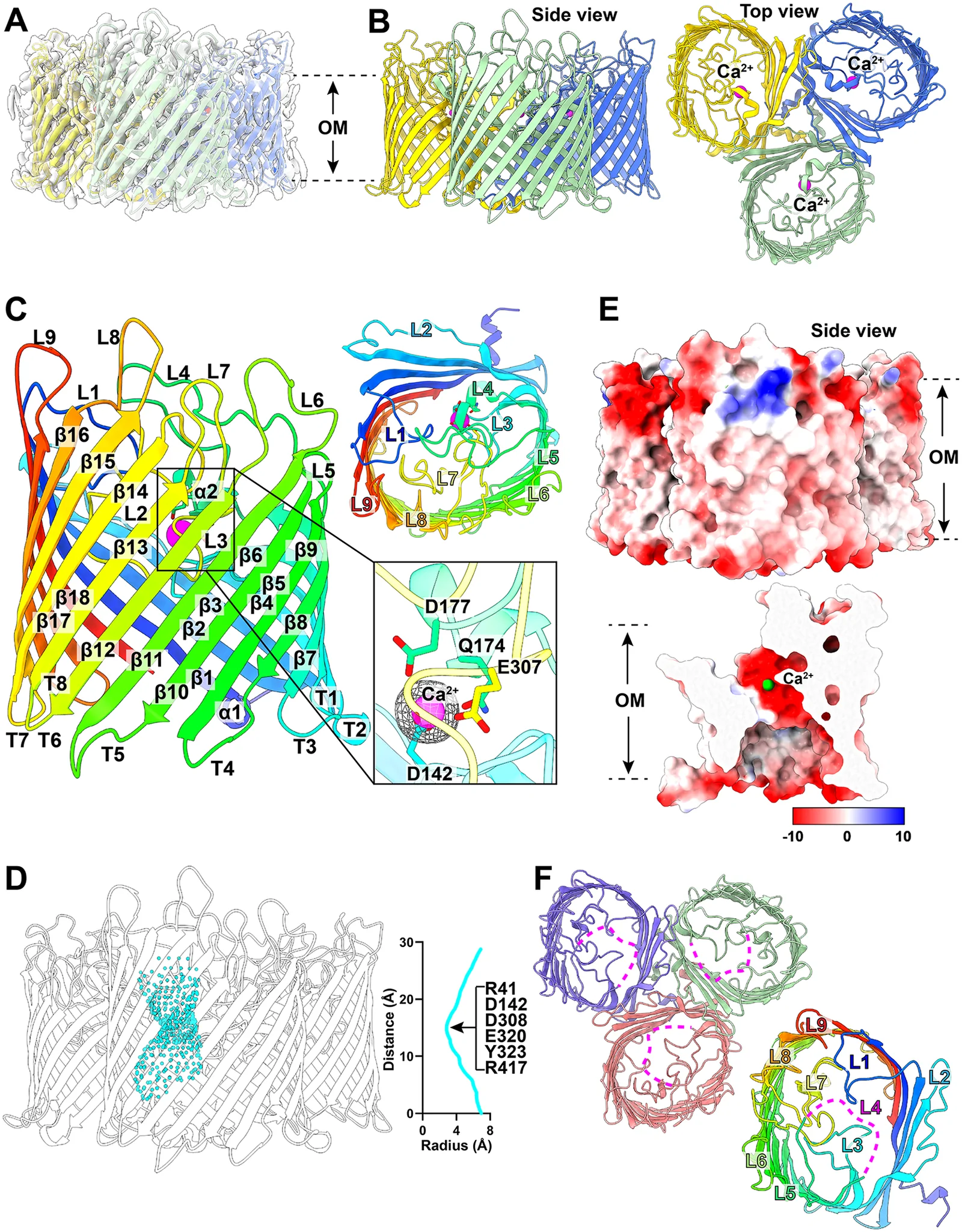
Structure of PorA. **(A)** Side view of the cryo-EM map of trimeric PorA-I at a resolution of 2.47 Å. The final structural model of the complex is included. **(B)** Side and top views of ribbon diagrams of the structure of trimeric PorA-I at this resolution. In **(A)** and **(B)**, individual protomers of PorA-I are colored green, yellow, and blue. **(C)** Side and top views of ribbon diagrams of the structure of a PorA-I protomer. The secondary structural elements of PorA are labeled. The bound Ca^2+^ ion is represented by a magenta sphere. The flexible loops L3, L4 and L7 fold into the middle of the pore to participate in channel formation. The cryo-EM density of bound Ca^2+^ is in gray meshes. Residues D142, Q174, D177, and E307 participating in Ca^2+^ binding are depicted as sticks. **(D)** The PorA channel. The channel, calculated by HOLE (http://www.holeprogram.org), is indicated by blue dots. The central hole of the 18-stranded β-barrel of each PorA-I protomer generates a continuous channel that facilitates substrate transport across the OM. The narrow region of the channel is surrounded by residues R41, D142, D308, E320, Y323, and R417. **(E)** Electrostatic surface potentials of trimeric PorA-I and a channel formed by a PorA-I protomer. The blue (>10 *k*_B_*T*) and red (<−10 *k*_B_*T*) colors of the electrostatic surface potential indicate the positively and negatively charged areas of the protein, respectively, where *k*_B_ is the Boltzmann constant and *T* is absolute temperature. White denotes the area between 10 *k*_B_*T* and −10 *k*_B_*T*. The calculations clearly depict the negatively charged nature of the channel formed by a PorA-I protomer. The bound Ca^2+^ ion is colored green. **(F)** Top view of the structure of the PorA-II trimer at a resolution of 2.74 Å. The three PorA-II protomers are colored salmon, slate, and green. A top view of the structure of a protomer of PorA-II is also included, indicating that the flexible loop L4 is missing in the structure. 2D classes and final reconstructions of PorA-I and PorA-II are illustrated in S1 Fig. The secondary structural topology of PorA-I is indicated in S2 Fig. Structural refinement statistics of PorA-I and PorA-II are shown in S2 Table.

*C. jejuni* typically colonizes within the gut of the host, but a recently emerged hypervirulent clone has shown to be capable of moving across the intestinal track, leading to bacteremia and abortion in pregnant animals [27]. Through the methodology of “directed genome evolution” [27], followed by selection in an animal disease model and whole-genome sequence analysis, it was found that mutations of the PorA membrane protein are the main cause of these abortions [27]. These results also highlight that PorA is a key virulence factor and an attractive candidate for the development of therapeutic strategies to combat *Campylobacter* infections. Site-specific mutagenesis indicates that amino acid substitutions on L4 of PorA are required to establish the abortion phenotype of *C. jejuni* [27]. Our cryo-EM structure of PorA-I depicts that L4 is a surface-exposed loop likely responsible for direct interaction with the host. In fact, of the 18 mutations observed in PorA of the hypervirulent SA strain, nine of these substitutions were found within the flexible L4. Subsequent experiments have also verified that only mutations in L4 were needed to cause abortion in guinea pig models [27].

***Structure of PorA-II.*** The second subclass contains 50,557 single particles, which allowed us to solve the structure of this subclass, designated as PorA-II, to a resolution of 2.74 Å (Figs 2F and S1; S2 Table). The overall structure of PorA-II is very similar to that of PorA-I with an r.m.s.d. of 0.4 Å when these two structures are superimposed (S4 Fig). The structure of PorA-II indicates that this protein also exists as a homotrimer, where each PorA protomer presents as an 18-stranded β-barrel protein. When the structures of PorA-I and PorA-II are compared, the major difference arises from the flexible surface-exposed loop L4. In PorA-I, residues 171–177 of L4 constitute a short α2 helix, which appears to be important for Ca^2+^ recognition with Q174 and D177 participating in ion binding. In PorA-II, the cryo-EM densities originating from residues 171–191, which comprise the majority of L4, are missing (Figs 2F and S4). In addition, no bound Ca^2+^ ion is found in this cryo-EM structure. It appears that the secondary structural elements of L4 may not be stable and are likely unstructured in the absence of bound Ca^2+^. This observation indeed highlights the intimate coordination between PorA and Ca^2+^. It is known that host Ca^2+^ ions can trigger bacterial virulence, adhesion, host cellular damage, and host-defense resistance [44]. In addition, it has been demonstrated that the host intracellular Ca^2+^ concentration is a key factor to regulate *Campylobacter* virulence, where *C. jejuni* is capable of mobilizing the host’s intracellular Ca^2+^ to initiate an invasion into host intestinal cells [45]. To that end, our structures suggest that PorA may be able to participate in Ca^2+^ signaling to manipulate host cells and facilitate *C. jejuni* invasion.

### OMP50/CjTK

*C. jejuni* OMP50 is an OM porin with a size of approximately 50 kDa [29]. Interestingly, this membrane protein is a species-specific porin expressed in *C. jejuni* and *C. lari* [32,46]. Its gene *cj1170c* is not ubiquitous in *Campylobacter* spp. OMP50 is homologically very distinct from other gram-negative OMPs of similar sizes. It caught the attention of the scientific community when it was revealed that this membrane protein is the only *C. jejuni* tyrosine kinase (CjTK) and also a major virulence factor of the bacterium [30]. Thus, it appears that OMP50 is also a moonlighting protein, serving as both an OM porin and a tyrosine kinase, where phosphotyrosine signaling modifies several outer membrane and periplasmic proteins required for bacterial capsule and polysaccharide synthesis [30]. Therefore, disruption of OMP50 signaling may be a promising direction to pursue in order to deliver a novel antimicrobial therapy to treat this infection.

***Structure of OMP50-I.*** Our GENTLE approach allowed us to obtain the first structure of the OMP50 membrane protein. We collected a total of 30,483 single particles for this class of images. Like PorA, this class of particles can be divided into two subsets with similar structural features, representing two distinct conformations of this OMP. We accumulated 21,652 single particles of the first subset of images, and then constructed the cryo-EM map to a resolution of 2.82 Å (S1 Fig and S2 Table). The program DeepTracer [35] led us to identify this protein as full-length OMP50. We then solved its structure, designated as OMP50-I, to this resolution (Fig 3A–3C). The structure reveals that the architecture of OMP50-I is very distinct from that of PorA-I. Our data shows that OMP50-I is trimeric in form. Each OMP50-I protomer assembles in such a way that it creates a pore. Therefore, the trimer contains three separate pores. Each OMP50-I protomer contains two α-helices and 16 β-strands that are folded into a 16-stranded β-barrel (β1–β16) with a central pore (Figs 3C and S5). Some of these β-strands, such as β4-β6, are quite short in length and can only span part of the OM. Each OMP50 protomer also constitutes eight extracellular loops (L1–L8) and seven periplasmic loops (T1–T7) that link the 16 β-strands to create the core of the β-barrel. Six of these flexible L loops, including L2–L7, are rather long, containing more than 20 amino acids. Surprisingly, our cryo-EM structure reveals that the N-terminal end of each OMP50-I protomer forms an elongated α-helix (α1) of 44 residues. Within the trimer, the three α1 helices intimately interact with each other through coiled-coil interactions to assemble an antenna-like three-helix bundle (Fig 3A–3E). The vertical dimension of this helix bundle is approximately 60 Å long, protruding into the periplasm from the periplasmic surface of the OM. Based on the structural information, this elongated periplasmic bundle may be critical for OMP50-I signaling from the outside to the inside of the bacterium. The trimeric structure of OMP50-I suggests that the linker region (residues 77–89) between α1 and β1, the transmembrane β-strands, β2-β4, and the flexible L loops, L2 and L4, create the trimeric interface.

**Fig 3:**
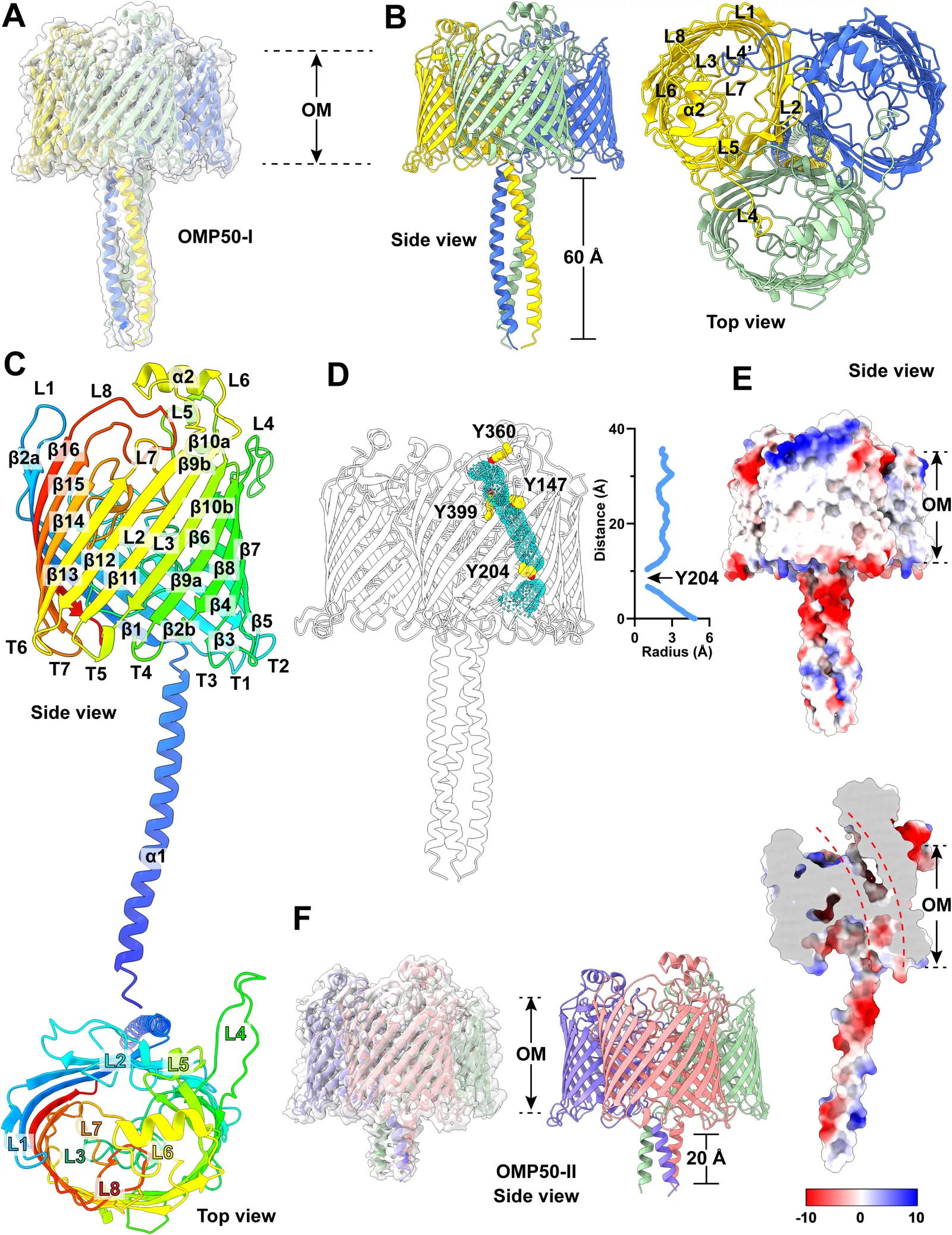
Structure of OMP50. **(A)** Side view of the cryo-EM map of trimeric OMP50-I at a resolution of 2.82 Å. The final structural model is included. **(B)** Ribbon diagrams of side and top views of the structure of trimeric OMP50-I at this resolution. In **(A)** and **(B)**, individual protomers of OMP50-I are colored green, yellow and blue. The top view of the structure of OMP50-I clearly indicates that L4′ of the blue protomer stretches into the center of the pore of the yellow protomer, contributing to the formation of the pore of the yellow protomer. **(C)** Ribbon diagrams of side and top views of the structure of an OMP50-I protomer, showing that the OMP50-I protomer contains two α helices and 16 β-strands that are folded into an OM domain of 16-stranded β-barrel and a periplasmic domain containing a single N-terminal α helix. The flexible loops L3, L6, L7, and L8 are found to fold into the middle of the pore to form a valve-like feature. **(D)** The OMP50-I channel. The channel, calculated by HOLE (http://www.holeprogram.org), is indicated by blue dots. The central hole of the 16-stranded β-barrel of each OMP50-I protomer generates a channel, which is closed by residue Y204. The four tyrosine residues, Y147, Y204, Y360, and Y399, which line the wall of this channel, are colored yellow. **(E)** Electrostatic surface potentials of trimeric OMP50-I and a channel formed by an OMP50-I protomer. The blue (>10 *k*_B_*T*) and red (<−10 *k*_B_*T*) colors of the electrostatic surface potential indicate the positively and negatively charged areas of the protein, respectively, where *k*_B_ is the Boltzmann constant and *T* is absolute temperature. White denotes the area between 10 *k*_B_*T* and −10 *k*_B_*T*. The calculations depict that the channel is quite hydrophobic in nature. **(F)** Cryo-EM map and ribbon diagrams of the side view of the trimeric OMP50-II structure at a resolution of 2.81 Å. The final structural model is included in the cryo-EM map. Individual protomers of OMP50-II are colored salmon, slate and green. 2D classes and final reconstructions of OMP50-I and OMP50-II are illustrated in S1 Fig. The secondary structural topology of OMP50-I is indicated in S5 Fig. Structural refinement statistics of OMP50-I and OMP50-II are shown in S2 Table.

As a tyrosine kinase, each protomer of OMP50-I possesses 27 tyrosine residues. In *C. jejuni*, it was detected that tyrosine phosphorylation only occurs at the OM [30]. Our structure indeed indicates that these tyrosines are located at the β barrels of the OM region of OMP50-I (S6 Fig). Most of these tyrosines are found within the β-strands, residing by the wall of the β barrel that forms a pore in the middle of each OMP50-I protomer. Therefore, many of them may be responsible for anchoring this kinase to the OM. However, some of these tyrosines should be critical in creating a network to facilitate phosphorylation signaling at the OM while the periplasmic α-helical bundle of the OMP50-I trimer may serve as a receiver to promote the transmission of signals to regulate the bacterium. Notably, residues Y147 of L2, Y204 of L3, Y360 of L6, and Y399 of L7 point toward the middle of the pore, where L3, L6 and L7 seemingly stretch into this pore to facilitate this structural arrangement (Fig 3C). Residues 349–358 of L6 create a surface-exposed α-helix (α2) which may be important for host–pathogen interactions. In addition, L4′ (the ′ indicates structural elements belonging to the next protomer) is also observed to stretch into the center of the pore of this protomer, contributing to the formation of this pore (Fig 3B). Interestingly, the internal wall of the pore, formed by each OMP50-I protomer, is lined by Y147, Y204, Y360 and Y399, creating a path for substrate transport (Fig 3D). The entire path of the pore is very narrow with an average radius of <3 Å, where Y204 forms a constriction site and completely closes this pore. Therefore, these four tyrosines may be critically important for tyrosine phosphorylation as well as controlling the pore opening and closing for substrate transport. Indeed, it has been reported that a mutation of Y147 to a phenylalanine abolished this kinase’s function [30]. Like PorA, electrostatic surface potential calculations depict that the extracellular surface of the OMP50-I β-barrel is predominantly neutral, but residues at locations corresponding to both surfaces of the OM create positively and negatively charged patches to facilitate the anchoring of this membrane protein at the OM (Fig 3E). In addition, the internal pore formed by each β barrel of the OMP50-I protomer is mostly neutral in charge, quite distinct from the pore formed by the PorA protomer. Due to the neutrality of the inner pore combined with the arrangement of the four tyrosines (Y147, Y204, Y360 and Y399) lining the pore, one would expect OMP50 to be more favorable in shuttling neutral and/or positively charged ligands across the OM. Indeed, it has been documented that OMP50 is capable of forming a cation-selective pore with a major conductance of 50–60 pS [29].

As mentioned, the transmembrane region of trimeric OMP50-I forms three identical pores, similar to those found in PorA and other trimeric porins such as OmpF and OmpC [19]. However, a detailed inspection of the architecture of the three OMP50-I pores indicates that the trimeric organization of OMP50-I is very distinct from other porins. Residues 240–260 of each OMP50-I protomer form an elongated loop L4 (Fig 3B, top view). This random loop protrudes into the center of the β barrel of the next OMP50-I protomer, constituting part of the central pore of the next protomer and likely also participating in the opening and closing of this pore. Based on the structural information, the three pores of the OMP50-I trimer are presumed to function in a dependent manner, where they coordinate with each other to control substrate transport across the OM.

***Structure of OMP50-II.*** We next investigated the second subset consisting of 8,831 single particles of this OMP50 class of images (S1 Fig). The constructed cryo-EM map allowed us to obtain a 2.81-Å resolution cryo-EM map (Figs 3F and S1). The map clearly indicated that these particles represent images of the OMP50 protein. However, the cryo-EM density corresponding to the N-terminal end of the periplasmic α1-helical bundle of the structure cannot be clearly seen. Densities corresponding to these helices can be observed when lowering the contour level of the cryo-EM map (S7 Fig), indicating that the conformation of this helical bundle extending into the periplasmic space may be quite flexible in nature. We then solved its cryo-EM structure, designated as OMP50-II, to this resolution to obtain its detailed structural information.

Similar to the structure of full-length OMP50 (OMP50-I) indicated above, OMP50-II exists as a homotrimer, where each OMP50-II protomer within the trimer is identical in conformation. In comparison with OMP50-I and OMP50-II, the conformations of the OM domains of these two structures are nearly identical, where superimposition of the OM domains of these two OMP50 trimers results in an r.m.s.d. of 0.3 Å (S8A Fig). As indicated, it appears that the major difference between these two structures arises from the N-terminal α-helical coiled-coil domain coordinated by helix α1 in the periplasm (S8A Fig). In OMP50-I, a full-length trimeric antenna-like three-helix bundle made up of helix α1 (residues 36–79) from each OMP50-I protomer is established in the middle of the trimer. In OMP50-II, only the C-terminal portion of α1 (residues 63–79) forming 1/3 of the length of this trimeric antenna can be easily seen (Fig 3F), as the cryo-EM densities originating from residues 36–62 comprising 2/3 of the length of this antenna are difficult to trace from the cryo-EM map. It is possible that these residues are very flexible and likely unstructured. The flexibility of these structural elements at the N-terminal end of the antenna-like feature may be important for facilitating phosphotyrosine signaling. It should be noted that our structure of OMP50-II resembles the published structure of the *Veillonella parvula* OmpM trimer [47], where only 16 residues from each protomer of the periplasmic α-helical domain can be seen in the cryo-EM structures. Superimposition of the structures of trimeric *C. jejuni* OMP50-II and *V. parvula* OmpM (PDB ID: 8YBS) [47] gives rise to an r.m.s.d. of 7.7 Å (S8B Fig), indicating that the structures of these two proteins are very different from each other.

### Cj0034c/CjSLP

In addition to PorA and OMP50, we identified a third class of images. We collected a total of 9,242 single particles and resolved a cryo-EM map of this class of images to a resolution of 3.13 Å (Figs 4A–4C and S1; S2 Table). The program DeepTracer [35] allowed us to reveal the identity of this membrane protein as Cj0034c, also called CjSLP, a known adhesion factor [48]. One of the key steps required to establish a successful *C. jejuni* infection is adhesion to the host cell. As Cj0034c appears to be a critical virulence factor, it has been identified as an attractive vaccine candidate [49]. Cj0034c has also been found to be immunogenic, and an antigen of Cj0034c has been observed to provide a significant protection against invasion in the spleen and liver [49].

**Fig 4:**
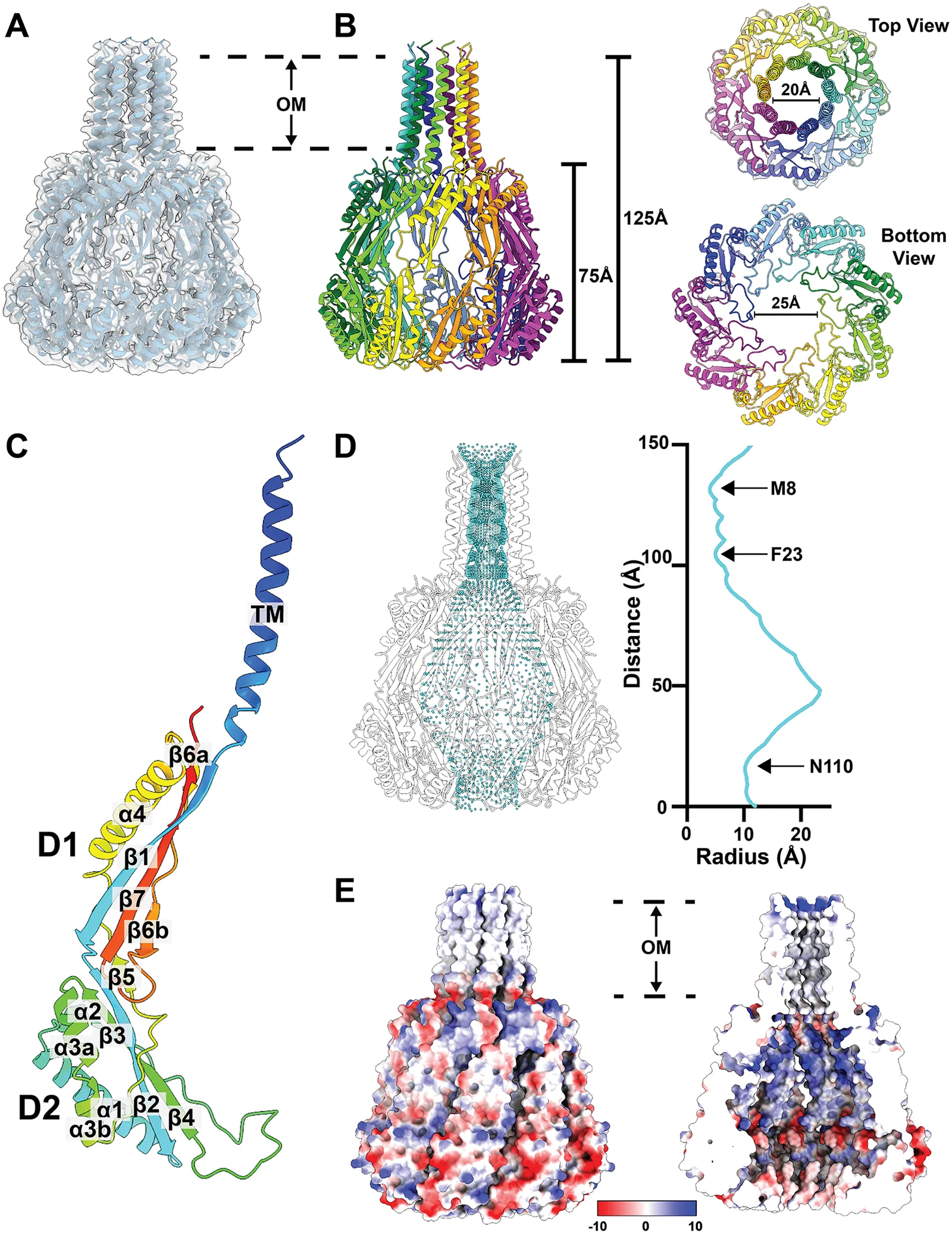
Structure of Cj0034c. **(A)** Side view of the cryo-EM map of nonameric Cj0034c at a resolution of 3.13 Å. The final structural model is included. **(B)** Side, top, and bottom views of ribbon diagrams of the Cj0034c nonamer at this resolution. **(C)** Ribbon diagrams of the structure of a Cj0034c protomer. The secondary structural elements are indicated. **(D)** The Cj0034c channel. The nonameric Cj0034c channel spans the membrane and part of the periplasmic space. This channel, calculated by HOLE (http://www.holeprogram.org), is indicated by blue dots. **(E)** Electrostatic surface potentials of the full-length Cj0034c nonamer. The blue (>10 *k*_B_*T*) and red (<−10 *k*_B_*T*) colors of the electrostatic surface potential indicate the positively and negatively charged areas of the protein, respectively, where *k*_B_ is the Boltzmann constant and *T* is absolute temperature. White denotes the area between 10 *k*_B_*T* and −10 *k*_B_*T*. The calculations provide charge distributions of the outermost surface and the interior of the channel of the Cj0034c nonamer. Overall, the interior of the nonameric channel is positively charged. 2D class and the final reconstruction of Cj0034c are illustrated in S1 Fig. The secondary structural topology of Cj0034c is indicated in S9 Fig. Structural refinement statistics of Cj0034c are shown in S2 Table.

Our cryo-EM structure allowed us to reveal for the first time the full-length architecture of Cj0034c and its assembly in native *C. jejuni* membranes. Nine protomers of Cj0034c stack side-by-side to form a doorknob-like apparatus (Fig 4A and 4B). Each protomer of Cj0034c consists of a transmembrane domain and a periplasmic domain (Figs 4C and S9). The N-terminal residues 6–34 form a single α-helical transmembrane helix (TM) that spans the entire thickness of the membrane. The majority of the amino acids, 36–230, are folded into a large periplasmic domain which can be further divided into two subdomains (D1 and D2) (Figs 4C and S9). Subdomain D1 is located right next to the TM and is composed of four β-sheets and one α-helix, whereas subdomain D2 constitutes five β-sheets and two α-helices. Different domains and subdomains are connected by flexible linkers, which also form hinges between them. Our cryo-EM structure of the periplasmic domain of each Cj0034c protomer is in good agreement with the X-ray structure of the monomeric soluble Cj0034c protein containing only D1 and D2 [48]. Superimposition of the periplasmic domain of our cryo-EM structure to the X-ray structure of Cj0034c (PDB ID: 7C51) [48] gives rise to an r.m.s.d. of 1.0 Å (S10 Fig).

It should be noted that Cj0034c was initially reported to be an OMP, but the N-terminal signaling peptide and outer membrane-spanning sequence were subsequently cleaved before protein maturation [21,50]. Therefore, the mature protein was presumed to become a soluble periplasmic protein, similar to the *Brucella* BP26/OMP28 homologous protein [48,49]. Surprisingly, our cryo-EM structure indicates that Cj0034c is an integral membrane protein possessing a single α-helical TM of approximately 50 Å long that spans the entire membrane thickness. The periplasmic domain of the nonameric protein creates a cage-like feature with dimensions of 75 Å long and 95 Å wide, making the entire oligomeric protein resemble a doorknob. The central core of the Cj0034c nonamer creates an open channel at both ends that directly connects both sides of the membrane of the bacterium (Fig 4D). The internal diameters of this channel at the two membrane surfaces are 20 Å and 25 Å, respectively (Fig 4B). Electrostatic surface potential calculations show that the internal wall of the nonameric Cj0034c channel is positively charged in nature (Fig 4E), suggesting that Cj0034c may be more favorable to bind negatively charged or neutral substrates.

In gram-negative bacteria, based upon all current reported structures, the IMP and OMP classes are distinct from each other. IMPs use α-helical structural features and OMPs utilize β-stranded secondary elements to constitute their TM domains. According to this structural information, the full-length Cj0034c membrane protein likely forms a typical IMP with a single-α-helical TM from each protomer to span the IM. However, recently the crystal structure of an atypical mycobacterial OMP MctB (Rv1698) has been reported [51]. The structure of this mycobacterial membrane protein shows an α-helical membrane-spanning region across the outer mycomembrane, where the N-terminal signaling peptide has dual functions. It guides the expressed protein to the outer mycomembrane as well as creates an α-helical TM domain [51]. Based on the structural information of MctB, it appears that Cj0034c could represent an unusual gram-negative OMP possessing all α-helical secondary structural elements in the TM domain. If this is the case, then the N-terminal sequence of Cj0034c may also hold a dual purpose. It could lead the protein to target the OM as well as participate in bridging the OM with an all α-helical TM domain, similar to that found in MctB. Therefore, the structure of the Cj0034c nonamer may exemplify the first structure of a gram-negative OMP featuring with an α-helical TM domain. Further studies are necessary to determine the spatiotemporal nature of this membrane protein as well as its complete physiological functions.

## Discussion

The *C. jejuni* OMPs are considered to be major virulence factors of the bacterium. They are crucial for adhesion and colonization to the host, scavenging nutrients, and transmitting signals from the outside to the inside of the bacterial cell. In this study, we show that our GENTLE cryo-EM methodology is capable of simultaneously targeting and solving structures of OMPs, including PorA or MOMP, OMP50 or CjTK, and Cj0034c or CjSLP, from crude, native *C. jejuni* membranes. These three proteins are critical virulence factors important for adhesion, host–pathogen interactions, and/or signal transduction [27,30,49]. They are also potential targets for the development of novel antibacterial strategies to combat *C. jejuni* infection [27,30,49]. Notably, this approach allowed us to obtain the first structural information of the OMP50 kinase, and the first structure of the full-length Cj0034c adhesin. Our methodology also led us to visualize the natural oligomerization states of these membrane proteins in native *C. jejuni* membranes. Cj0034c has been implicated as an attractive vaccine target, as it was found to be an important adhesion factor to host epithelial cells [49]. Its homologous protein BP26/OMP28 of *Brucella* spp. has been used for vaccine development to combat Brucellosis [52,53]. The mature BP26 protein is produced by cleaving its OM segment and assembling into a cage-like periplasmic protein complex composed of 16 protomers [54]. As such, the final protein product of Cj0034c was thought to reside in the periplasm after proteolysis, with the outer membrane-spanning sequence truncated to form the soluble protein as seen in the X-ray structure [48]. Surprisingly, our cryo-EM structure indicates that full-length Cj0034c is not a periplasmic protein. Instead, it is a membrane protein containing nine Cj0034c protomers that form a nonameric channel, which spans the lipid bilayer and part of the periplasmic space of *C. jejuni*. Each protomer of Cj0034c constitutes a single TM α-helix and a large periplasmic domain. It is not entirely clear if full-length Cj0034c spans the IM or OM to function. Whether Cj0034c is an IMP or OMP must await further experimental confirmation. Based on the structural information, Cj0034c likely forms a typical membrane protein that spans the IM of the bacterium. If Cj0034c indeed traverses across the OM instead, then this protein would represent the first known gram-negative OMP with its N-terminal amino acids forming both the signaling peptide and the α-helical structural elements that allow it to span the OM rather than residues that would be cleaved off after maturation as proposed [21,48,50].

Our cryo-EM structure of OMP50 offers the first glance of the architecture of this trimeric channel protein. OMP50 is a two-domain protein, consisting of an all β-stranded transmembrane domain and an all α-helical periplasmic domain. Interestingly, our structure depicts that all 27 tyrosines from each protomer of the trimer are localized at the OM of *C. jejuni*. Which of these tyrosines are critical for phosphorylation and signaling has yet to be determined. It seems that the secondary structure of the periplasmic domain of OMP50 can be quite flexible, capable of switching from all α-helical (as seen in OMP50-I) to mostly unstructured elements (as seen in OMP50-II). It has been shown that reactive oxygen species (ROS) released from the intestinal mucosa of the host upon *C. jejuni* invasion inhibit this phosphotyrosine network. This results in the inactivation of UDP-GlcNAc/Glc 4-epimerase (Gne), causing altered polysaccharide synthesis [30]. Therefore, OMP50 is critically important for signaling host–pathogen interactions, particularly in establishing an infection. Our approach leading to the structure of OMP50 has provided the first glimpse of this important tyrosine network. Further investigations targeting these tyrosines will help us understand how *C. jejuni* interacts with its host.

PorA is known to associate with the virulence and pathogenicity of *C. jejuni* [27,55,56]. Our cryo-EM structures of PorA depict that loop L4, facing the outside of the bacterial cell, is highly flexible in nature and critically important for Ca^2+^ binding. Interestingly, it has been demonstrated that L4 of PorA is responsible for hypervirulence of the *C. jejuni* SA strain [27], where mutations on this loop cause systemic infection and abortion in pregnant animals. The fact that L4 is directly related to Ca^2+^ binding and virulence suggests that the host intracellular Ca^2+^ concentration may be a key factor to regulate *Campylobacter* virulence. If this is the case, then PorA may be an important player for Ca^2+^ signaling to prime bacterial virulence. In addition to virulence and Ca^2+^, PorA has been directly linked to antibiotic resistance [57–59]. Mutations on L4 and other flexible loops of PorA have been found to mediate carbapenem resistance in *C. jejuni* [57,59]. It has been suggested that mutations in PorA and overexpression of β-lactamase are two major mechanisms for *Campylobacter* to confer resistance to carbapenems [57]. Therefore, a detailed understanding of the molecular mechanisms underlying PorA virulence and function may open a novel avenue for treating *Campylobacter* infections.

We believe that our GENTLE cryo-EM methodology can be generally applied to study many different membrane protein systems within their respective cell type. The procedures of sucrose cushion, *in silico* purification, sorting of images, and retrieving particles are all aimed to enrich protein particle populations in order to accumulate a large enough data set for high-resolution cryo-EM structural determinations. Therefore, this methodology may not be limited to the study of membrane proteins with high abundance. It may also be applicable for the investigation of membrane proteins with modest populations embedded in their respective membranes. As we study these target proteins in a more native environment, it is expected that these proteins will be more stable and thereby more amenable for structural study in these conditions. In addition, it should be preferable for protein protomers to assemble in their natural oligomerization state, which in turn would facilitate structural-functional studies. For instance, our GENTLE approach provides us the first insight into the structure, function, and oligomerization of Cj0034c, where nine protomers of Cj0034c assemble as a nonameric channel, directly spanning a layer of the bacterial membrane. Additionally, we believe that our GENTLE methodology can be applied to the study of membrane protein complex systems, where different protein components may be more favorable to form a fully assembled complex within their native environment, which in turn would facilitate structural determination. This would negate the need to use cross-linking or other protein modification procedures to determine assembled complex structures. Therefore, our GENTLE methodology is expected to be quite powerful for the study of large membrane protein complexes that span the cell envelope in both prokaryotic and eukaryotic cell types, opening the door for drug development targeting unique, previously neglected complex systems.

## Methods

### Cell growth, membrane preparation, and cryo-EM sample preparation

*C. jejuni* NCTC 11168 were grown in 3 l of Mueller-Hinton (MH) broth at 42 °C under microaerobic conditions (85% N_2_, 10% CO_2_, and 5% O_2_) for 16 h with shaking at 120 rpm. Cells were harvested by centrifugation at 12,000*g* for 10 min. The collected bacteria were resuspended in buffer containing 20 mM Tris-HCl (pH 7.5), 50 mM magnesium acetate, 1 mM dithiothreitol (DTT) and 0.5 mM ethylenediaminetetraacetic acid (EDTA) then lysed using a French pressure cell. The membrane fraction was collected and washed two times with buffer containing 100 mM sodium phosphate (pH 7.2) and 10% glycerol, and then two times with buffer containing 20 mM HEPES-NaOH (pH 7.5) and 20 mM NaCl. The membrane was then solubilized in 1% (w/v) lauryl maltose neopentyl glycol (LMNG) overnight at 4 °C. Insoluble material was removed by ultracentrifugation at 186,000*g* for 40 min.

The membrane protein component of the solubilized membrane was enriched using sucrose cushion ultracentrifugation. Briefly, 1 ml of sucrose buffer A (60% sucrose, 20 mM Na-HEPES (pH 7.5), 20 mM NaCl and 0.001% LMNG) was added to the bottom of the centrifugation tube, and then 1 ml of sucrose buffer B (15% sucrose, 20 mM Na-HEPES (pH 7.5), 20 mM NaCl and 0.001% LMNG) was carefully added on top of the first layer. These sucrose concentrations were empirically optimized: the 60% cushion forms a dense barrier that retains membrane protein complexes above the tube bottom, while the 15% layer allows membrane proteins to migrate through but restricts entry of excess free detergent micelles. Next, a 2 ml detergent-solubilized sample was slowly added to the top of the sucrose layers and centrifuged at 100,000 g for 4 h at 4 °C. After centrifugation, the lower half of the 15% sucrose fraction, which is enriched in membrane protein complexes while depleted of free detergent, was carefully collected. It was then diluted 8-fold with buffer containing 20 mM HEPES-NaOH (pH 7.5), 20 mM NaCl, and 0.001% LMNG to reduce the sucrose concentration and facilitate subsequent pelleting of the enriched membrane proteins. The diluted fraction was then centrifuged at 100,000*g* for 16 h at 4°C. Subsequently, the protein pellet was dissolved with 10 µl of a buffer containing 20 mM HEPES-NaOH (pH 7.5), 20 mM NaCl and 0.001% LMNG. This protein solution was then centrifuged at 20,000*g* for 2 min to remove insoluble particles. The same buffer was used to adjust the protein concentration of the enriched sample to 10 mg/ml (estimated using a NanoDrop OneC instrument, Thermo Fisher Scientific).

For cryo-EM sample preparation, 2.5 μl of enriched membrane sample was directly applied to glow-discharged holey carbon grids (Quantifoil Cu R1.2/1.3, 300 mesh). The optimal blotting time was determined to be 12 s. After blotting, the sample was plunge-frozen in liquid ethane using a Vitrobot (Thermo Fisher Scientific). The resulting grids were then transferred into cartridges prior to data collection.

### Data collection and data processing

Samples were collected at the Cleveland Center for Membrane & Structural Biology (CCMSB) using a Titan Krios cryo-electron transmission microscope (Thermo Fisher Scientific) operating at 300 kV, equipped with a BioQuantum energy filter (Gatan) with a slit width of 20 eV. A total of 19,448 movies were collected using SerialEM for automated data acquisition. Images were recorded at 81,000× magnification using a K3 direct electron detector (Gatan) in super-resolution mode, with a defocus range of 0.8–1.5 μm. Each movie consisted of 40 frames with a total electron dose ranging from 41 to 50 e^−^/Å^2^, corresponding to a super-resolution pixel size of 0.535 Å/pixel and a physical pixel size of 1.07 Å/pixel (S2 Table). Initial image stacks were binned by a factor of 2 and motion corrected using cryoSPARC v3 [60]. The patchCTF function was used to estimate the contrast transfer function [60]. The Topaz tool [61], default ResNet16 (64 U) pre-trained model, was used to pick initial particle sets from 19,448 movies, yielding 29,498,465 particles. The BaR protocol [33] was used to separate initial structure classes. During the “build” phase, the particles were sorted via multiple rounds of 2D classification to identify particles with distinct structural features. Particle stacks consisting of distinct 2D representations were each subjected to independent ab initio reconstruction, and 3 starting target models with structurally viable global features and 3 random unfeatured decoy models were then used as filters in a series of heterogeneous refinements using the original particle stack. At the “retrieve” phase, each target model retrieved additional particles relative to the initial stack, including a more diverse set of orientations. These refined particle stacks were then used to generate refined 3D models. These models were used as updated filters to retrieve additional particles belonging to each distinct class. This BaR cycle was repeated iteratively until no additional particles were incorporated and no further improvement in map quality was observed, at which point three distinct particle populations were identified: 309,511 particles, 35,032 particles and 26,588 particles. Subsequently, 3D classification was performed on each population independently to sort different conformations, non-uniform refinement and local refinement was used on the final 3D models to generate high-resolution density maps. Protein identification of the PorA, OMP50, and Cj0034c membrane proteins were done by a combination of manual building and utilizing the program DeepTracer [35]. The presence of these three membrane proteins in our sample was also confirmed by proteomics (S1 Table).

### Model building and refinement

Based on their respective cryo-EM maps, model building of all proteins were performed using Coot [36]. Structural refinements were accomplished using the phenix.real_space_refine program [62] implemented in the PHENIX suite [37]. The final atomic models of these membrane proteins were evaluated using MolProbity [63]. The statistics associated with data collection, 3D reconstruction and model refinement are included in S2 Table.

### Proteomic analysis

A 1× lysis buffer (5% SDS in 50 mM tiethylammonium bicarbonate (TEAB)) was added into 10 μg of the membrane sample to a final volume of 46 μl. The sample was digested with a trypsin solution (0.1 μg/μl in 50 mM TEAB) and transferred onto the S-trap spin column. A Bruker TimsTof Pro2 Q-Tof LC-MS system operating in positive ion mode coupled with a CaptiveSpray ion source (both from Bruker Daltonik GmbH, Bremen) and a Bruker 15 cm x 75 μm id C18 ReproSil AQ, 1.9 μm, 120 Å reversed-phase capillary chromatography column was used for peptide analysis. Five μl extract was injected, and the peptides were eluted from the column by an acetonitrile/0.1% formic acid gradient at a flow rate of 0.3 μl/min. The digests were analyzed using a Parallel Accumulation–Serial Fragmentation (PASEF) DDA method [64] to select precursor ions for fragmentation with a TIMS-MS scan followed by 10 PASEF MS/MS scans. The TIMS-MS survey scan was acquired between 0.60 and 1.6 Vs/cm^2^ and 100–1,700 *m*/*z* with a ramp time of 166 ms. The total cycle time for the PASEF scans was 1.2 seconds and the MS/MS experiments were performed with collision energies between 20 eV (0.6 Vs.cm^2^) to 59 eV (1.6 Vs/cm^2^). Precursors with 2–5 charges were selected with the target value set to 20,000 a.u. and the intensity threshold set to 2,500 a.u. Precursors were dynamically excluded for 0.4 s.

The DDA data acquired by TimsTofs (Bruker) were converted into.mgf files using AlphaTims [65], an open-source package developed by Mann lab, before analyzing using the program Proteome Discoverer 2.4 (Thermo Fisher Scientific).

## Supporting information

S1 Fig Native *C. jejuni* membrane sample cryo-EM data processing workflow. A total of 19,448 micrographs were collected and processed through motion correction to extract 29,498,465 particles. Following the build and retrieve (BaR) computational pipeline, including 2D classification, multi-class ab initio reconstruction, and heterogeneous refinement, particles were sorted into three major populations. Representative 2D class averages are shown for each population. Subsequent 3D classification and non-uniform refinement with local refinement yielded structures of multiple membrane proteins. Final reconstructions include: PorA-I at 2.47 Å resolution, PorA-II at 2.74 Å resolution, OMP50-I at 2.82 Å resolution, OMP50-II at 2.81 Å resolution, and Cj0034c at 3.13 Å resolution.
(TIF)

S2 Fig Secondary structural topology of PorA. The topology diagram was generated based on the cryo-EM structure of PorA-I. The outer membrane (OM) is colored yellow. The transmembrane β-strands are shown as green arrows. The α-helices located outside the membrane are colored magenta.
(TIF)

S3 Fig Structural comparison of *C. jejuni* PorA-I with the published X-ray structure. Superimposition of the PorA-I structure from this study (green) with the previously reported structure (PDB: 5LDT, purple). The inset shows a detailed view of the calcium binding site. Key residues involved in Ca^2+^ coordination are shown as sticks with residue numbers corresponding to this study. The Ca^2+^ ion is shown as a green sphere.
(TIF)

S4 Fig Structural comparison of *C. jejuni* PorA-I and PorA-II. Side and top views showing superimposition of the PorA-I (yellow) with PorA-II (magenta) structures. Loop 2 (L2) and loop 4 (L4) are indicated with black arrows. The Ca^2+^ion in the PorA-I structure is shown as a green sphere. L4 of PorA-II is missing in the structure (black dotted curve).
(TIF)

S5 Fig Secondary structural topology of OMP50. The topology diagram was generated based on the cryo-EM structure of OMP50. The outer membrane (OM) is colored yellow. The transmembrane β-strands are shown as green arrows. The α-helices located outside the membrane are colored magenta.
(TIF)

S6 Fig The tyrosine network of OMP50. **(A)** Side and top views of the OMP50-I trimer. **(B)** Side and top views of a OMP50-I protomer. In (A) and (B), all of the tyrosines are shown as yellow sticks.
(TIF)

S7 Fig Cryo-EM maps of OMP50-II at different contour levels. This figure includes the side view of the final structure of the OMP50-II trimer, and the side view of cryo-EM maps of OMP50-II contoured at 4.6 σ, 3.3 σ and 2.0 σ levels. These maps indicate that the N-terminal residues 21–62 are likely to be flexible.
(TIF)

S8 Fig Structural comparison of *C. jejuni* OMP50-I, *C. jejuni* OMP50-II and *V. parvula* OmpM. **(A)** Side view of the superimposition of the structures of OMP50-I (cyan) and OMP50-II (yellow). This superimposition gives rise to an r.m.s.d. of 0.3 Å. **(B)** Side view of the superimposition of the structures of OMP50-II (green) and *V. parvula* OmpM (red). This superimposition gives rise to an r.m.s.d. of 7.7 Å, suggesting that the structures of *C. jejuni* OMP50-II and *V. parvula* OmpM are very different from each other.
(TIF)

S9 Fig Secondary structural topology of Cj0034c. The topology diagram was generated based on the cryo-EM structure of Cj0034c. The outer membrane (OM) is colored yellow. The transmembrane α-helix (TM) is colored blue. The secondary structural elements of the periplasmic subdomains D1 and D2 are colored red and purple.
(TIF)

S10 Fig Structural comparison of the periplasmic domain of *C. jejuni* Cj0034c with the published X-ray structure containing only the periplasmic domain. Side view showing superimposition of the Cj0034c structure (blue) with a published *C. jejuni* Simpl-like protein structure (PDB ID: 7C51) (white).
(TIF)

S1 Table Native *C. jejuni* membrane protein proteomics data analysis of LC–MS/MS. (DOCX)

S2 Table Native *C. jejuni* membrane protein cryo-EM data collection and refinement statistics. (DOCX)

## Abbreviations

BaR: Build-and-Retrieve
CCMSB: Cleveland Center for Membrane & Structural Biology
CDC: Centers for Disease Control and Prevention
CjTK: *C. jejuni* tyrosine kinase
DTT: dithiothreitol
EDTA: ethylenediaminetetraacetic acid
GDN: glycol-diosgenin
GENTLE: Gradient Enrichment of Native Targets from Lipid Environments
IM: inner membrane
IMPs: inner membrane proteins
LMNG: lauryl maltose neopentyl glycol
MOMP: major outer membrane protein
OM: outer membrane
OMPs: outer membrane proteins
PASEF: Parallel Accumulation–Serial Fragmentation
PorA: porous protein A
ROS: reactive oxygen species
SA: sheep abortion
TEAB: tiethylammonium bicarbonate
TM: transmembrane helix

## References

[1] Fischer F, Wolters D, Rögner M, Poetsch A. Toward the complete membrane proteome: high coverage of integral membrane proteins through transmembrane peptide detection. Mol Cell Proteomics. 2006;5(3):444–53. doi: 10.1074/mcp.M500234-MCP200 16291997

[2] von Heijne G. Recent advances in the understanding of membrane protein assembly and structure. Q Rev Biophys. 1999;32(4):285–307. doi: 10.1017/s0033583500003541 11194567

[3] Cavalier-Smith T. Membrane heredity and early chloroplast evolution. Trends Plant Sci. 2000;5(4):174–82. doi: 10.1016/s1360-1385(00)01598-3 10740299

[4] Casadio R, Fariselli P, Finocchiaro G, Martelli PL. Fishing new proteins in the twilight zone of genomes: the test case of outer membrane proteins in *Escherichia coli* K12, *Escherichia coli* O157:H7, and other Gram-negative bacteria. Protein Sci. 2003;12(6):1158–68. doi: 10.1110/ps.0223603 12761386 PMC2323886

[5] Ruiz-Palacios GM. The health burden of *Campylobacter* infection and the impact of antimicrobial resistance: playing chicken. Clin Infect Dis. 2007;44(5):701–3. doi: 10.1086/509936 17278063

[6] van Doorn PA, Ruts L, Jacobs BC. Clinical features, pathogenesis, and treatment of Guillain-Barré syndrome. Lancet Neurol. 2008;7(10):939–50. doi: 10.1016/S1474-4422(08)70215-1 18848313

[7] CDC. *Campylobacter* (Campylobacteriosis). 2021.

[8] Moffatt CRM, Kennedy KJ, O’Neill B, Selvey L, Kirk MD. Bacteraemia, antimicrobial susceptibility and treatment among *Campylobacter*-associated hospitalisations in the Australian Capital Territory: a review. BMC Infect Dis. 2021;21(1):848. doi: 10.1186/s12879-021-06558-x 34419003 PMC8379883

[9] Hussein K, Raz-Pasteur A, Shachor-Meyouhas Y, Geffen Y, Oren I, Paul M, et al. *Campylobacter* bacteraemia: 16 years of experience in a single centre. Infect Dis (Lond). 2016;48(11–12):796–9. doi: 10.1080/23744235.2016.1195916 27320494

[10] Korman TM, Varley CC, Spelman DW. Acute hepatitis associated with *Campylobacter jejuni* bacteraemia. Eur J Clin Microbiol Infect Dis. 1997;16(9):678–81. doi: 10.1007/BF01708559 9352262

[11] Ezpeleta C, de Ursua PR, Obregon F, Goñi F, Cisterna R. Acute pancreatitis associated with *Campylobacter jejuni* bacteremia. Clin Infect Dis. 1992;15(6):1050. doi: 10.1093/clind/15.6.1050 1457641

[12] Pope JE, Krizova A, Garg AX, Thiessen-Philbrook H, Ouimet JM. *Campylobacter* reactive arthritis: a systematic review. Semin Arthritis Rheum. 2007;37(1):48–55. doi: 10.1016/j.semarthrit.2006.12.006 17360026 PMC2909271

[13] Kosek M, Bern C, Guerrant RL. The global burden of diarrhoeal disease, as estimated from studies published between 1992 and 2000. Bull World Health Organ. 2003;81(3):197–204. 12764516 PMC2572419

[14] Ventola CL. The antibiotic resistance crisis: part 1: causes and threats. P T. 2015;40(4):277–83. 25859123 PMC4378521

[15] Craig M. CDC’s antibiotic resistance threats report, 2019. 2019.

[16] Nikaido H. Molecular basis of bacterial outer membrane permeability revisited. Microbiol Mol Biol Rev. 2003;67(4):593–656. doi: 10.1128/MMBR.67.4.593-656.2003 14665678 PMC309051

[17] Vaara M. Agents that increase the permeability of the outer membrane. Microbiol Rev. 1992;56(3):395–411. doi: 10.1128/mr.56.3.395-411.1992 1406489 PMC372877

[18] Saxena D, Maitra R, Bormon R, Czekanska M, Meiers J, Titz A, et al. Tackling the outer membrane: facilitating compound entry into Gram-negative bacterial pathogens. NPJ Antimicrob Resist. 2023;1(1):17. doi: 10.1038/s44259-023-00016-1 39843585 PMC11721184

[19] Masi M, Pagès J-M. Structure, function and regulation of outer membrane proteins involved in drug transport in enterobactericeae: the OmpF/C - TolC case. Open Microbiol J. 2013;7:22–33. doi: 10.2174/1874285801307010022 23569467 PMC3617542

[20] Kline KA, Dodson KW, Caparon MG, Hultgren SJ. A tale of two pili: assembly and function of pili in bacteria. Trends Microbiol. 2010;18(5):224–32. doi: 10.1016/j.tim.2010.03.002 20378353 PMC3674877

[21] Parkhill J, Wren BW, Mungall K, Ketley JM, Churcher C, Basham D, et al. The genome sequence of the food-borne pathogen *Campylobacter jejuni* reveals hypervariable sequences. Nature. 2000;403(6770):665–8. doi: 10.1038/35001088 10688204

[22] Flanagan RC, Neal-McKinney JM, Dhillon AS, Miller WG, Konkel ME. Examination of *Campylobacter jejuni* putative adhesins leads to the identification of a new protein, designated FlpA, required for chicken colonization. Infect Immun. 2009;77(6):2399–407. doi: 10.1128/IAI.01266-08 19349427 PMC2687328

[23] Konkel ME, Garvis SG, Tipton SL, Anderson DE Jr, Cieplak W Jr. Identification and molecular cloning of a gene encoding a fibronectin-binding protein (CadF) from *Campylobacter jejuni*. Mol Microbiol. 1997;24(5):953–63. doi: 10.1046/j.1365-2958.1997.4031771.x 9220003

[24] Rivera-Amill V, Kim BJ, Seshu J, Konkel ME. Secretion of the virulence-associated *Campylobacter* invasion antigens from *Campylobacter jejuni* requires a stimulatory signal. J Infect Dis. 2001;183(11):1607–16. doi: 10.1086/320704 11343209

[25] Moser I, Schroeder W, Salnikow J. *Campylobacter jejuni* major outer membrane protein and a 59-kDa protein are involved in binding to fibronectin and INT 407 cell membranes. FEMS Microbiol Lett. 1997;157(2):233–8. doi: 10.1111/j.1574-6968.1997.tb12778.x 9435102

[26] Ferrara LGM, Wallat GD, Moynié L, Dhanasekar NN, Aliouane S, Acosta-Gutiérrez S, et al. MOMP from *Campylobacter jejuni* is a trimer of 18-stranded β-barrel monomers with a Ca2+ ion bound at the constriction zone. J Mol Biol. 2016;428(22):4528–43. doi: 10.1016/j.jmb.2016.09.021 27693650 PMC5090048

[27] Wu Z, Periaswamy B, Sahin O, Yaeger M, Plummer P, Zhai W, et al. Point mutations in the major outer membrane protein drive hypervirulence of a rapidly expanding clone of *Campylobacter jejuni*. Proc Natl Acad Sci U S A. 2016;113(38):10690–5. doi: 10.1073/pnas.1605869113 27601641 PMC5035848

[28] Bolla JM, Loret E, Zalewski M, Pagés JM. Conformational analysis of the *Campylobacter jejuni* porin. J Bacteriol. 1995;177(15):4266–71. doi: 10.1128/jb.177.15.4266-4271.1995 7543469 PMC177172

[29] Bolla JM, Dé E, Dorez A, Pagès JM. Purification, characterization and sequence analysis of Omp50,a new porin isolated from *Campylobacter jejuni*. Biochem J. 2000;352 Pt 3(Pt 3):637–43. doi: 10.1042/bj3520637 11104668 PMC1221499

[30] Corcionivoschi N, Alvarez LAJ, Sharp TH, Strengert M, Alemka A, Mantell J, et al. Mucosal reactive oxygen species decrease virulence by disrupting *Campylobacter jejuni* phosphotyrosine signaling. Cell Host Microbe. 2012;12(1):47–59. doi: 10.1016/j.chom.2012.05.018 22817987 PMC3749511

[31] Dedieu L, Pagès J-M, Bolla J-M. Environmental regulation of *Campylobacter jejuni* major outer membrane protein porin expression in *Escherichia coli* monitored by using green fluorescent protein. Appl Environ Microbiol. 2002;68(9):4209–15. doi: 10.1128/AEM.68.9.4209-4215.2002 12200267 PMC124132

[32] Dedieu L, Pagès J-M, Bolla J-M. Use of the omp50 gene for identification of *Campylobacter* species by PCR. J Clin Microbiol. 2004;42(5):2301–5. doi: 10.1128/JCM.42.5.2301-2305.2004 15131219 PMC404618

[33] Su C-C, Lyu M, Morgan CE, Bolla JR, Robinson CV, Yu EW. A “Build and Retrieve” methodology to simultaneously solve cryo-EM structures of membrane proteins. Nat Methods. 2021;18(1):69–75. doi: 10.1038/s41592-020-01021-2 33408407 PMC7808410

[34] Zhang Z, Maharjan R, Gregor WD, Klenotic PA, Yu EW. Structures of MmpL complexes reveal the assembly and mechanism of this family of transporters. Sci Adv. 2025;11(33):eadx1129. doi: 10.1126/sciadv.adx1129 40802754 PMC12346298

[35] Pfab J, Phan NM, Si D. DeepTracer for fast de novo cryo-EM protein structure modeling and special studies on CoV-related complexes. Proc Natl Acad Sci U S A. 2021;118(2):e2017525118. doi: 10.1073/pnas.2017525118 33361332 PMC7812826

[36] Emsley P, Cowtan K. Coot: model-building tools for molecular graphics. Acta Crystallogr D Biol Crystallogr. 2004;60(Pt 12 Pt 1):2126–32. doi: 10.1107/S0907444904019158 15572765

[37] Adams PD, Grosse-Kunstleve RW, Hung LW, Ioerger TR, McCoy AJ, Moriarty NW, et al. PHENIX: building new software for automated crystallographic structure determination. Acta Crystallogr D Biol Crystallogr. 2002;58(Pt 11):1948–54. doi: 10.1107/s0907444902016657 12393927

[38] Dé E, Jullien M, Labesse G, Pagès JM, Molle G, Bolla JM. MOMP (major outer membrane protein) of *Campylobacter jejuni*; a versatile pore-forming protein. FEBS Letters. 2000;469(1):93–7. 10.1016/s0014-5793(00)01244-8 10708763

[39] Mahdavi J, Pirinccioglu N, Oldfield NJ, Carlsohn E, Stoof J, Aslam A, et al. A novel O-linked glycan modulates *Campylobacter jejuni* major outer membrane protein-mediated adhesion to human histo-blood group antigens and chicken colonization. Open Biol. 2014;4(1):130202. doi: 10.1098/rsob.130202 24451549 PMC3909276

[40] Cody AJ, Maiden MJC, Dingle KE. Genetic diversity and stability of the porA allele as a genetic marker in human *Campylobacter* infection. Microbiology (Reading). 2009;155(Pt 12):4145–54. doi: 10.1099/mic.0.031047-0 19744989 PMC2885669

[41] Sahin O, Plummer PJ, Jordan DM, Sulaj K, Pereira S, Robbe-Austerman S, et al. Emergence of a tetracycline-resistant *Campylobacter jejuni* clone associated with outbreaks of ovine abortion in the United States. J Clin Microbiol. 2008;46(5):1663–71. doi: 10.1128/JCM.00031-08 18322054 PMC2395063

[42] Sahin O, Fitzgerald C, Stroika S, Zhao S, Sippy RJ, Kwan P, et al. Molecular evidence for zoonotic transmission of an emergent, highly pathogenic *Campylobacter jejuni* clone in the United States. J Clin Microbiol. 2012;50(3):680–7. doi: 10.1128/JCM.06167-11 22189122 PMC3295108

[43] Dhanasekar NN, Aliouane S, Winterhalter M, Pagès J-M, Bolla J-M. Peptide translocation across MOMP, the major outer membrane channel from *Campylobacter jejuni*. Biochem Biophys Rep. 2017;11:79–83. doi: 10.1016/j.bbrep.2017.06.007 28955771 PMC5614690

[44] King MM, Kayastha BB, Franklin MJ, Patrauchan MA. Calcium regulation of bacterial virulence. Adv Exp Med Biol. 2020;1131:827–55. doi: 10.1007/978-3-030-12457-1_33 31646536 PMC7473484

[45] Hu L, Raybourne RB, Kopecko DJ. Ca2+ release from host intracellular stores and related signal transduction during *Campylobacter jejuni* 81-176 internalization into human intestinal cells. Microbiology (Reading). 2005;151(Pt 9):3097–105. doi: 10.1099/mic.0.27866-0 16151220

[46] Dedieu L, Pagès J-M, Bolla J-M. The omp50 gene is transcriptionally controlled by a temperature-dependent mechanism conserved among thermophilic *Campylobacter* species. Res Microbiol. 2008;159(4):270–8. doi: 10.1016/j.resmic.2008.03.002 18485677

[47] Silale A, Zhu Y, Witwinowski J, Smith RE, Newman KE, Bhamidimarri SP, et al. Dual function of OmpM as outer membrane tether and nutrient uptake channel in diderm Firmicutes. Nat Commun. 2023;14(1):7152. doi: 10.1038/s41467-023-42601-y 37932269 PMC10628300

[48] Oh HB, Yoon S-I. Structural analysis of a Simpl-like protein from *Campylobacter jejuni*. Biochem Biophys Res Commun. 2020;529(2):270–6. doi: 10.1016/j.bbrc.2020.05.211 32703422

[49] Nielsen LN, Luijkx TA, Vegge CS, Johnsen CK, Nuijten P, Wren BW, et al. Identification of immunogenic and virulence-associated *Campylobacter jejuni* proteins. Clin Vaccine Immunol. 2012;19(2):113–9. doi: 10.1128/CVI.05161-11 22155767 PMC3272937

[50] Gundogdu O, Bentley SD, Holden MT, Parkhill J, Dorrell N, Wren BW. Re-annotation and re-analysis of the *Campylobacter jejuni* NCTC11168 genome sequence. BMC Genomics. 2007;8:162. doi: 10.1186/1471-2164-8-162 17565669 PMC1899501

[51] Siroy A, Sun D, Paul A, Rowland JL, Jones LM, Prevelige P, et al. Oligomerization is required for channel formation of MctB and for copper resistance of *Mycobacterium tuberculosis*. J Biol Chem. 2026;302(2):111037. doi: 10.1016/j.jbc.2025.111037 41380963 PMC12859517

[52] Gupta VK, Radhakrishnan G, Harms J, Splitter G. Invasive *Escherichia coli* vaccines expressing *Brucella melitensis* outer membrane proteins 31 or 16 or periplasmic protein BP26 confer protection in mice challenged with *B. melitensis*. Vaccine. 2012;30(27):4017–22. doi: 10.1016/j.vaccine.2012.04.036 22546330 PMC3361596

[53] Clapp B, Walters N, Thornburg T, Hoyt T, Yang X, Pascual DW. DNA vaccination of bison to brucellar antigens elicits elevated antibody and IFN-γ responses. J Wildl Dis. 2011;47(3):501–10. doi: 10.7589/0090-3558-47.3.501 21719815 PMC9828159

[54] Kim D, Park J, Kim SJ, Soh Y-M, Kim HM, Oh B-H, et al. *Brucella* immunogenic BP26 forms a channel-like structure. J Mol Biol. 2013;425(7):1119–26. doi: 10.1016/j.jmb.2013.01.015 23353825

[55] Dai L, Wu Z, Xu C, Sahin O, Yaeger M, Plummer PJ. The Rho-independent transcription terminator for the porA gene enhances expression of the major outer membrane protein and *Campylobacter jejuni* virulence in abortion induction. Infect Immun. 2019;87(12):e00687-19. 10.1128/IAI.00687-19 PMC6867836 31570559

[56] Elmi A, Nasher F, Dorrell N, Wren B, Gundogdu O. Revisiting Campylobacter jejuni Virulence and Fitness Factors: Role in Sensing, Adapting, and Competing. Front Cell Infect Microbiol. 2021;10:607704. doi: 10.3389/fcimb.2020.607704 33614526 PMC7887314

[57] Maurille C, Guérin F, Jehanne Q, Audemard-Verger A, Isnard C, Verdon R, et al. Occurrence of in vivo carbapenem-resistant Campylobacter coli mediated by porA point mutation and overexpression of bla(OXA-489) under meropenem treatment. Clin Microbiol Infect. 2024;30(11):1478–80. 10.1016/j.cmi.2024.06.027 38969103

[58] Zhuo R, Younes RL, Ward K, Yang S. Carbapenem resistant *Campylobacter jejuni* bacteremia in a Bruton’s X-linked agammaglobulinemia patient. Eur J Clin Microbiol Infect Dis. 2024;43(12):2459–63. doi: 10.1007/s10096-024-04937-1 39316322 PMC11608308

[59] Nunes A, Oleastro M, Alves F, Liassine N, Lowe DM, Benejat L, et al. Recurrent *Campylobacter jejuni* infections with in vivo selection of resistance to macrolides and carbapenems: molecular characterization of resistance determinants. Microbiol Spectr. 2023;11(4):e0107023. doi: 10.1128/spectrum.01070-23 37358443 PMC10434052

[60] Punjani A, Rubinstein JL, Fleet DJ, Brubaker MA. cryoSPARC: algorithms for rapid unsupervised cryo-EM structure determination. Nat Methods. 2017;14(3):290–6. doi: 10.1038/nmeth.4169 28165473

[61] Bepler T, Morin A, Rapp M, Brasch J, Shapiro L, Noble AJ, et al. Positive-unlabeled convolutional neural networks for particle picking in cryo-electron micrographs. Nat Methods. 2019;16(11):1153–60. doi: 10.1038/s41592-019-0575-8 31591578 PMC6858545

[62] Afonine PV, Poon BK, Read RJ, Sobolev OV, Terwilliger TC, Urzhumtsev A, et al. Real-space refinement in PHENIX for cryo-EM and crystallography. Acta Crystallogr D Struct Biol. 2018;74(Pt 6):531–44. doi: 10.1107/S2059798318006551 29872004 PMC6096492

[63] Chen VB, Arendall WB 3rd, Headd JJ, Keedy DA, Immormino RM, Kapral GJ, et al. MolProbity: all-atom structure validation for macromolecular crystallography. Acta Crystallogr D Biol Crystallogr. 2010;66(Pt 1):12–21. doi: 10.1107/S0907444909042073 20057044 PMC2803126

[64] Cox J, Neuhauser N, Michalski A, Scheltema RA, Olsen JV, Mann M. Andromeda: a peptide search engine integrated into the MaxQuant environment. J Proteome Res. 2011;10(4):1794–805. doi: 10.1021/pr101065j 21254760

[65] Willems S, Voytik E, Skowronek P, Strauss MT, Mann M. AlphaTims: indexing trapped ion mobility spectrometry-TOF data for fast and easy accession and visualization. Mol Cell Proteomics. 2021;20:100149. doi: 10.1016/j.mcpro.2021.100149 34543758 PMC8526765

