# Supplementary material for: Structural insights into the outer membrane proteins PorA, OMP50 and Cj0034c from native Campylobacter jejuni membranes
Source: PLoS Biol. 2026 Aug 28;24(8):e3003961. doi: 10.1371/journal.pbio.3003961 (PMC13759207; doi:10.1371/journal.pbio.3003961)
Supplement: Supplementary file 1 [file pbio.3003961.s011.docx]

| **Table S1. Native *C. jejuni* membrane protein proteomics data analysis of LC-MS/MS.** | | | | | |
| --- | --- | --- | --- | --- | --- |
| **Ranking** | **Protein ID** | **Mass**  **(kDa)** | **Pep**  **All** | **Seq.**  **Cov**  **(%)** | **Sequest**  **Score** |
| 1 | **Major outer membrane protein (P80672)** | 45.7 | 39 | 76 | 14848.90 |
| 2 | Formate dehydrogenase large subunit (Selenocysteine containing) (Q0P8A7) | 103.9 | 100 | 81 | 4856.85 |
| 3 | Fumarate reductase flavoprotein subunit (Q0PBA1) | 73.7 | 90 | 84 | 4521.90 |
| 4 | nitrite reductase (cytochrome; ammonia-forming) (Q0P8Q2) | 69.2 | 75 | 72 | 3784.14 |
| 5 | Ubiquinol-cytochrome C reductase cytochrome C subunit (Q0P972) | 41.4 | 39 | 74 | 3259.95 |
| 6 | Ni/Fe-hydrogenase large subunit (Q0P8Z0) | 63.5 | 55 | 84 | 2424.25 |
| 7 | Cb-type cytochrome C oxidase subunit II (Q0P8C8) | 24.9 | 22 | 71 | 1626.28 |
| 8 | Ubiquinol-cytochrome C reductase iron-sulfur subunit (Q0P970) | 18.1 | 17 | 59 | 1415.80 |
| 9 | Fumarate reductase iron-sulfur subunit (Q0PBA0) | 27.5 | 24 | 63 | 1369.04 |
| 10 | NADH dehydrogenase I chain G (Q0P855) | 93.3 | 64 | 66 | 1278.07 |
| **⁝** |  |  |  |  |  |
| 14 | **50 kDa outer membrane protein (Q0P986)** | 53.3 | 33 | 67 | 1027.90 |
| **⁝** |  |  |  |  |  |
| 24 | **Periplasmic protein Cj0034c (Q0PC92)** | 26.4 | 24 | 81 | 768.35 |

Pep all: the total number of peptides mapped to the protein
