# Supplementary material for: Structural insights into the outer membrane proteins PorA, OMP50 and Cj0034c from native Campylobacter jejuni membranes
Source: PLoS Biol. 2026 Aug 28;24(8):e3003961. doi: 10.1371/journal.pbio.3003961 (PMC13759207; doi:10.1371/journal.pbio.3003961)
Supplement: Supplementary file 2 [file pbio.3003961.s012.docx]

**Table S2. Native *C. jejuni* membrane protein cryo-EM data collection and refinement statistics.**

| **Data collection** |  | | | | |
| --- | --- | --- | --- | --- | --- |
| Magnification | 81,000 | | | | |
| Voltage (kV) | 300 | | | | |
| Electron Microscope | Krios-GIF-K3 | | | | |
| Defocus (um) | -0.8 to -1.5 | | | | |
| Energy filter width (eV) | 20 | | | | |
| Pixel size (Å) | 0.535 | | | | |
| Total dose (e^-^/ Å^2^) | 50 | | 41.0 | | 50.0 |
| Number of frames | 40 | | 40 | | 40 |
| Number of micrographs | 7,176 | | 5,220 | | 7,052 |
| Number of initial particles | 29,498,465 | | | | |
| **Refinement** |  | | | | |
| Number of final particles | 85,621 | 50,557 | 21,652 | 8,831 | 9,242 |
|  | PorA-I | PorA-II | OMP50-I | OMP50-II | Cj0034c |
| GS-FSC Resolution (0.143, Å) | 2.47 | 2.74 | 2.82 | 2.81 | 3.13 |
| Symmetry | C3 | C3 | C3 | C3 | C9 |
| Model composition |  | |  | | |
| Chains | 4 | 3 | 3 | 3 | 9 |
| Protein residues | 1,206 | 1,143 | 1,314 | 1,233 | 2,079 |
| Ligand | 3 | 0 | 0 | 0 | 0 |
| r.m.s.d. |  | |  | | |
| Bond lengths (Å) | 0.004 (0) | 0.003 (0) | 0.002 (0) | 0.002 (0) | 0.003 (0) |
| Bond angles (°) | 0.507 (4) | 0.433 (10) | 0.458 (6) | 0.433 (3) | 0.440 (1) |
| Validation |  | |  | | |
| MolProbity score | 1.28 | 1.17 | 1.59 | 1.29 | 0.64 |
| Clash score | 2.27 | 2.80 | 5.71 | 4.33 | 0.39 |
| Ramachandran plot |  | |  | | |
| Favored (%) | 97.58 | 97.52 | 97.02 | 97.64 | 98.64 |
| Allowed (%) | 2.42 | 2.48 | 2.98 | 2.36 | 1.36 |
| Disallowed (%) | 0 | 0 | 0 | 0 | 0 |
| CC Mask | 0.83 | 0.81 | 0.78 | 0.80 | 0.83 |
