# Supplementary material for: Structural insights into the outer membrane proteins PorA, OMP50 and Cj0034c from native Campylobacter jejuni membranes
Source: PLoS Biol. 2026 Aug 28;24(8):e3003961. doi: 10.1371/journal.pbio.3003961 (PMC13759207; doi:10.1371/journal.pbio.3003961)
Supplement: Supplementary file 3 [file pbio.3003961.s015.docx]

We are submitting a revised manuscript entitled “Targeting outer membrane proteins (OMPs) directly from crude *Campylobacter jejuni* membranes for structural determination using the “Gradient Enrichment of Native Targets from Lipid Environments” (GENTLE) methodology” to PLoS Biology for publication. Three of the referees have raised several important points that we have now addressed in this revision.

In this revised manuscript, we have added an additional section showing new structural information for OMP50 (OMP50-II) that has 2/3 of the alpha-helical coiled-coil periplasmic domain missing from the cryo-EM structure. When comparing OMP50-I (full-length OMP50) and OMP50-II (truncated OMP50), these two structures suggest that the conformation of the structural elements making up the coiled-coil domain are very flexible, which may be related to the function of this protein.

We have made substantial changes and have extensively rewritten the revised manuscript. We have also added subtitles on each of the sections to highlight each protein structure. In addition, a step-by-step GENTLE procedure has been added to provide more details about this methodology.

Our GENTLE approach leads to the first structural information of OMP50 and the first full-length structure of Cj0034c. We believe that our GENTLE methodology is quite powerful in that it allows us to identify and solve structures of these OMPs from *C. jejuni* membranes without their overexpression and purification. For these reasons, we believe that this work is significant and will make an impact in the field.

All changes are highlighted with blue color in the markup manuscript.

Our point-by-point response to the referees’ comments follows this letter.

Thank you for your continued consideration.

Sincerely,

REVIEWS:


Reviewer #1 (Bert van den Berg, identifies himself): The manuscript by Yu and colleagues describes an application of the BaR methodology, where total membranes are isolated followed by cryo-EM structure determination of the most abundant/stable complexes. In this case the target organism is the Gram-negative pathogen Campylobacter jejuni, and the membranes are isolated via mild detergent (LMNG) extraction combined with sucrose gradient centrifugation. The authors are able to solve cryo-EM structures of three membrane proteins: PorA (or MOMP), Omp50 and Cj0034c. The first two are outer membrane proteins (OMPs) and the last one an unexpected inner membrane protein (IMP) of unknown function. The structures of PorA generate some extra, potentially important information that add on insights garnered from previous structures determined by X-ray crystallography, i.e. the role of calcium in the conformation of the functionally important loop L4. No previous structures were determined for Omp50, and the current structure identifies a trimer with a periplasmic coiled coil as well as narrow channels in each trimer that are lined by some tyrosine residues. Finally, the Cj0034c structure presents a major surprise since the structure suggests (but see comments below) that it forms a nonameric IM channel with a large periplasmic domain, which contrast with previous notions based on crystallography (soluble periplasmic protein).

Overall, this is an interesting application of the BaR method that was developed and previously published by the authors. The structures are beautiful and skillfully done, and the manuscript is overall reasonably well-written, but not very reviewer-friendly (see comment 1). My main issue with the paper, combined with the fact that PLOS Biology publication is sought, is that it represents a bit of "stamp collecting", where the methodology can be applied in a fairly straightforward way to any organism of interest to solve MP structures. There are no hypothesis being tested regarding any of the interesting biology of Campylobacter jejuni (e.g. the role of Omp50 in Tyr phosphorylation), despite the wealth of information that is provided by the structures. Besides this, I have some comments for consideration by the authors:

We appreciate this reviewer indicated that “The structures are beautiful and skillfully done” in the manuscript. We would like to point out that there is not much information reported in the literature for the *C. jejuni* Omp50 and Cj0034c proteins. There is only one paper showing that Omp50 is a phosphotyrosine kinase (Corcionivoschi et al. 2012. Cell Host Microbe 12:47-59), but the molecular mechanism of this protein and detailed information about its tyrosine network are lacking. Therefore, the high-resolution structural information of this protein is valuable to the field. In the case of Cj0034c, the only structural information can be found in the literature is it as a truncated and soluble protein (Oh & Yoon. 2020. Biochem Biophys Res Comm 529:270-276), which is monomeric in form. We suspect that this structure has a limitation in that the recombinant protein was a truncated protein to begin with, and the crystal lattices may limit the protein to only assemble as a monomer, which may not be physiologically relevant. Since we elucidate these membrane protein structures from crude, detergent-solubilized native membranes, we believe that our structures are more physiologically relevant as they were determined in a more native environment without recombinant protein overexpression and purification. Therefore, the scientific impact and discovery of this manuscript is highly significant for the field of *C. jejuni* physiology and virulence, and the information within can substantially move the field forward.

We believe that our manuscript presents substantial technical novelty, and the methodology is strongly hypothesis driven. We combine two very complimentary techniques, sucrose cushion to enrich protein molecules, and BaR to enhance single-particle population, to develop the GENTLE methodology. We hypothesize that via the processes of the enrichment of biomacromolecules, *in silico* purification and sorting of images, we can isolate and separate various classes of biomacromolecules, build high-resolution cryo-EM maps and simultaneously identify and solve cryo-EM structures of targeted membrane proteins in native membranes without recombinant protein overexpression and purification. We also hypothesize that this GENTLE methodology can be applied to obtain high-resolution structural information of *C. jejuni* outer membrane porins that are critical for virulence, pathogenesis, and host-pathogen interaction.

This work is definitely not part of a “stamp collection”, as we have a well-defined goal to focus on targeting outer membrane porins residing at the *C. jejuni* outer membranes. Indeed, we have demonstrated we can simultaneously solve structures of the major outer membrane porin PorA and minor outer membrane porin Omp50 from one single membrane sample, again without protein overexpression and purification. Based on the mass spectrometry data, our *C. jejuni* membrane sample is highly populated with inner membrane proteins. Table S1 of this manuscript indeed indicates that among the top 10 mostly populated proteins in our sample, nine of them are found to participate in forming inner membrane protein complexes. These include the Frd, Pet, and Hyd inner membrane protein complexes. It should be noted that our newly developed GENTLE methodology also allows us to solve high-resolution structures of these inner membrane protein complexes to high resolutions (see attached figure below). However, the goal of our research work is to elucidate the critical outer membrane proteins of *C. jejuni*, these inner membrane protein complexes are outside the scope of our research goal, and we did not report these structures here.

We also believe that this work is not “fairly straightforward”, as the method of sorting images into different protein classes from a very heterogeneous data embedded with many different proteins, building initial maps, retrieving data to search for more particles and improve the quality of maps, etc., are not trivial and need a lot of training. We hope that this reviewer would understand the difficulty of the nature of the work. Indeed, both Reviewers 2 and 3 very much appreciate our work. Reviewer 2 highlighted our work that “This MS describes the use of an exciting method for identification and solving of cryo-EM structures from bacterial cells without specific overexpression” and “This manuscript is chockful of insights about the three/four structurally characterized proteins.” In addition, Reviewer 3 indicated that “This is a strong paper that makes dual contributions to the field.”

1. There are no subheadings in the text. Also, line numbers are missing. Finally, it would be appreciated if (at least) the main figures with their legends are inserted within the text.

In this revised manuscript, we have made substantial changes and reformatted the style. The subheadings have been added, following this reviewer’s suggestion. We do not believe that PLoS Biol now requires us to insert main figures and legends in the text. Nonetheless, we have made a markup version of the manuscript that include figures and legends in the main text per this reviewer’s request.

2. On page 10, the authors say that the Omp50 architecture is "unique". In which way? 16-stranded beta-barrel trimers are every common, tyrosines also occur in large numbers in OMPs, and a helical coiled-coil was also recently reported in an OMP (PMID: 37932269). Please tone this down. There are also other instances where results and implications are overstated. Like I said above, it is a pity that the authors did not attempt to study more of the biology of this interesting protein now that they solved the structure. On page 11 it is implied that 27 tyrosine residues are a consequence of the function of the protein, without evidence being provided. Tyrosines are generally very abundant in OMPs.

We thank this reviewer to indicate this literature to us. This publication has been cited in this revised manuscript. The sentence “… unique among all currently available structures of OM porins or channels” has been removed from this revised manuscript. We actually solved two structures of Omp50, where one is in its full-length form and the other has 2/3 of the alpha-helical coiled-coil domain missing. We did not include the truncated structure in the original manuscript because we thought that the full-length structure would cover all of the information obtained from the truncated structure. However, this truncated structure is very similar to the structure of OmpM reported by this reviewer with a similar number of residues resolved in the coiled-coil stalk domain. Therefore, we decided to include a new section (Structure of OMP50-II) to report this truncated structure on pp. 16-17 of the revised version of the manuscript.

We would like to point out that did not intend to imply that all 27 tyrosine residues are a consequence of the function of the protein. What we believe is that the four tyrosines located in the middle of the pore may be crucial for the function of Omp50, and indeed one of these four tyrosines has been proven to be critical (Corcionivoschi et al. 2012. Cell Host Microbe 12:47-59). We have rephrased these sentences to “Most of these tyrosines are found within the β-strands, residing by the wall of the β barrel that forms a pore in the middle of each OMP50 protomer. Therefore, many of them may be responsible for anchoring this kinase to the OM. However, some of these tyrosines should be critical in creating a network to facilitate phosphorylation signaling at the OM …” on p. 13 of the revised manuscript.

3. The statement on page 12 at the end of the first paragraph is complete speculation. The same goes for the last sentence of the second paragraph. Also, the apparent fact that loop L4 forms part of the "valve" (is that the correct term?) of the neighbouring pore is not well shown.

The statement “Based on the structural information, it appears that the opening and closing of the pore, substrate transport, and tyrosine phosphorylation and signaling are likely coupled together in this membrane protein” has been removed and the term “valve” has also been removed.

4. Top page 13: is this a real Walker motif? Please show its location. Note also that the presence of ATP in any bacterial periplasmic space would not be expected.

We actually do not think that this is a real Walker motif, but it was previously reported in a published paper (Corcionivoschi et al. 2012. Cell Host Microbe 12:47-59). This paragraph has been removed from this revised manuscript.

5. The most puzzling of the new structures is that of Cj0034c. If it is IM localised/periplasmic, how could it be involved in adhesion and how could it be an attractive vaccine candidate? Or is this all based on faulty assumptions in previous studies (eg refs 42 and 43)? But if this protein is an IMP, the fairly large channel would present a serious breach of the IM barrier. It is very unlikely to "directly connect the periplasm with the cytoplasm", as stated on page 18. Is it possible that we are dealing with a highly unusual hydrophobic TM segment that is somehow inserted into the OM? The presence of an MFS type transporter within the operon (how do we know this?) would also suggest that this couples somehow to Cj0034c to form a channel across the periplasmic space (inserted into the OM). Has the gene been annotated correctly (is there an alternative start codon with a signal sequence). The open channel is just extremely unlikely to insert into the IM. The authors do not critically analyse the important implications of the structure and the previous literature. Finally, the structure by itself doesn't allow one to say conclusively whether a protein is IM or OM-localised.

Yes, this reviewer is correct. We are dealing with “a highly unusual hydrophobic TM segment that is somehow inserted into the OM”. It came across that we found a just published literature reporting an unusual mycobacterial outer membrane protein MctB (Siroy et al. 2026. J Biol Chem 302:111037), which utilizes an alpha helix to span the outer mycomembrane (similar to what we saw in the case of Cj0034c). Its N-terminal signaling peptide has dual functions, where it guides the expressed protein to the outer mycomembrane as well as creates an α-helical TM domain spanning the OM of the mycobacterium (Siroy et al. 2026. J Biol Chem 302:111037). Therefore, it is very likely that the signaling peptide of Cj0034c may also hold a dual-purpose to lead the protein to target the OM as well as participate in bridging the OM with an all α-helical TM domain.

A new paragraph “Based on the structural information, Cj0034c may represent an unusual OMP possessing all α-helical secondary structural elements in the TM domain. As all reported OMP structures of Gram-negative bacteria have the distinct signature of spanning the OM with β-stranded secondary elements, our structure of the Cj0034c nonamer may also exemplify the first structure of a Gram-negative OMP featuring with an α-helical TM domain. Recently, the crystal structure of an atypical mycobacterial OMP MctB (Rv1698) has been reported (Siroy et al. 2026. J Biol Chem 302:111037). The structure of this mycobacterial membrane protein also shows an α-helical membrane-spanning region across the outer mycomembrane, where the N-terminal signaling peptide has dual functions. It guides the expressed protein to the outer mycomembrane as well as creates an α-helical TM domain (Siroy et al. 2026. J Biol Chem 302:111037). It appears that the N-terminal sequence of Cj0034c may also hold a dual-purpose. It leads the protein to target the OM as well as participates in bridging the OM with an all α-helical TM domain, similar to that found in MctB.” has added on p. 18 of this revised manuscript to address this.

6. Page 17: rephrase "all 27 tyrosines, which are important for.......". What is then this network of tyrosines that the structure is purported to show, given that OMPs contain many Tyr residues?

We do not think all 27 tyrosines are important for signaling. However, we do think that the four tyrosines located in the middle of the pore may be critical for tyrosine phosphorylation and function. The statements have been rephrased to “Most of these tyrosines are found within the β-strands, residing by the wall of the β barrel that forms a pore in the middle of each OMP50 protomer. Therefore, many of them may be responsible for anchoring this kinase to the OM. However, some of these tyrosines should be critical in creating a network to facilitate phosphorylation signaling at the OM …” on p.13 of this revised manuscript.

7. The notion that GENTLE will be powerful for determination of fragile envelope-spanning complexes is not supported by the data. OMPs are known to be stable and the structure of Cj0034c also suggests it is very stable. If the others want to test this notion they should overexpress an efflux pump and see if they isolate a complex. This also brings me to the proteomics table: why are there no IMP complexes present within the data (or are there)? Is the method not as gentle as we are led to believe?

Our GENTLE methodology is designed to obtain structural information of membrane proteins from crude native membranes without overexpression and purification. As indicated at the beginning of responding to this reviewer’s comments, we have simultaneously identified and solved cryo-EM structures of three different inner membrane complexes. These three inner membrane proteins are the Frd, Pet, and Hyd complexes, which have been resolved to resolutions of 2.82, 3.03 and 2.77 Å, respectively (see attached figure). However, the goal of our research focuses on elucidating outer membrane proteins of *C. jejuni*, we did not report these structures as they are beyond the scope of our current work.

8. Methods: the section describing GENTLE is poor. It is important to include volumes of buffer, amount of membranes etc in order to ensure this can be repeated exactly. Also centrifuge used, tubes etc. After centrifugation, how is the membrane pellet dissolved exactly?

We have added step-by-step procedures of the GENTLE methodology in this revised manuscript. We thought that the procedures in the method section of the original manuscript were quite detailed. Nonetheless, we have carefully added related volumes, amounts, etc. (if they were missing from the original manuscript) into this revised manuscript. We have also added a few statements “After centrifugation, the membrane protein pellet at the bottom was carefully collected by removing the sucrose buffers. Subsequently, the protein pellet was dissolved 10 µl in a buffer containing 20 mM Na-HEPES (pH 7.5), 20 mM NaCl and 0.001% LMNG. This protein solution was then centrifuged at 20,000 x g for 2 min to remove insoluble particles.” on p. 23 of this revised manuscript to add a little more detailed information on sample preparation and the sucrose cushion procedure.

9. Proteomics: "10 ug of membrane sample". Based on BCA (proteins) or weight?

It was based on nanodrop. This was mentioned in the original manuscript as “(estimated using a NanoDrop OneC instrument, Thermo Fisher Scientific)”.

10. The main figures are quite fuzzy for some reason in the provided PDF.

All figures presented in the original manuscript are at high resolution, and we do not understand why the figures are fuzzy. Nonetheless, we have enhanced the resolution of these figures in the revised manuscript.


Reviewer #2: This MS describes the use of an exciting method for identification and solving of cryo-EM structures from bacterial cells without specific overexpression. The authors use the method to solve 3-4 structures of membrane proteins connected to the virulence of C. jejuni. This manuscript is chockful of insights about the three/four structurally characterized proteins.

We very much appreciate this reviewer’s comments indicating that “This MS describes the use of an exciting method for identification and solving of cryo-EM structures from bacterial cells without specific overexpression” and “This manuscript is chockful of insights about the three/four structurally characterized proteins.”

Although I see that this MS has already gone through one round of revision, I have not seen the explanation of changes and did not review the initial submission.

This is the first submission. It could be that the initial submission was missing something and got bound back for additional information.

A few questions and requests for improvement:

1) An important insight of this manuscript is that the GENTLE methodology allowed for identification and solving of cryo-EM structures of OMPs from native C. jejuni membranes without overexpression. And that this method is not restricted to proteins of high abundance. This point would be better illustrated if a table with relative abundances of the proteins solved as well as maybe some abundances of higher and lower abundance proteins to better understand the un-restrictedness and/or limitations of this method.

This methodology should not be restricted to proteins of high abundance. However, based on the mass spectrometry data (Table S1 (Table S2 of the original manuscript)), the ranking of the population of PorA, OMP50, and Cj0034c are at positions 1, 14, and 24 (24 is actually quite low), we decided to remove the statement “even though their natural expression levels are modest” (in the Abstract) and rephrase the statement to “It may be applicable to investigate membrane proteins with modest populations embedded in their respective membranes” (p. 21) to it tone down in this revised manuscript.

2) There is quite a bit of repetition in the section about the characterization of CjSLP. It reads like that section was written twice but one of the versions was never removed. Could these two sections be merged more effectively?

We have been very careful to proofread and edit this section of the revised manuscript to avoid repetition. Thank you for pointing this out.

3) What does the SLP of CjSLP stand for?

It stands for “*C. jejuni* signaling molecule that interacts with mouse pelle-like kinase (Simp)-like protein”. This was indicated on p. 4 of the original manuscript (p. 6 of this revised manuscript).

4) How is it clear that CjSLP is an inner membrane protein and not an unusual helical protein in the outer membrane?

We are so excited to find the just published crystal structure of an atypical mycobacterial OMP MctB (Rv1698) (Siroy et a. 2026. J Biol Chem 302:111037), which showed an α-helical membrane-spanning region similar to what we have seen in Cj0034c/CjSLP. Therefore, our cryo-EM structure of Cj0034c may represent the first example of a Gram-negative OMP featuring with an α-helical TM domain.

A new paragraph “Based on the structural information, Cj0034c may represent an unusual OMP possessing all α-helical secondary structural elements in the TM domain. As all reported OMP structures of Gram-negative bacteria have the distinct signature of spanning the OM with β-stranded secondary elements, our structure of the Cj0034c nonamer may also exemplify the first structure of a Gram-negative OMP featuring with an α-helical TM domain. Recently, the crystal structure of an atypical mycobacterial OMP MctB (Rv1698) has been reported (Siroy et al. 2026. J Biol Chem 302:111037). The structure of this mycobacterial membrane protein also shows an α-helical membrane-spanning region across the outer mycomembrane, where the N-terminal signaling peptide has dual functions. It guides the expressed protein to the outer mycomembrane as well as creates an α-helical TM domain (Siroy et al. 2026. J Biol Chem 302:111037). It appears that the N-terminal sequence of Cj0034c may also hold a dual-purpose. It leads the protein to target the OM as well as participates in bridging the OM with an all α-helical TM domain, similar to that found in MctB.” has added on p. 18 of this revised manuscript to address this.

5) The first paragraph of the introduction is misleading in two ways:
a. Yes, 20-30% of proteins are membrane proteins but that's all inner membrane/plasma membrane proteins. The outer membrane proteins that this MS describes are 1 -2 % of the proteins of Campylobacter.

Yes, this reviewer is correct. A few sentences “These membrane proteins can be classified into two distinct categories: α-helical and β-barrel transmembrane proteins. The α-helical transmembrane proteins are commonly found in plasma membranes of eukaryotic cells and inner membranes (IMs)/cytoplasmic membranes of bacterial cells, whereas the β-barrel transmembrane proteins are exclusively observed in outer membranes (OMs) of mitochondria, chloroplasts, and Gram-negative bacteria. In contrast to the α-helical IM proteins (IMPs), which constitute the majority of bacterial membrane proteins, β-barrel OM proteins (OMPs) comprise less than 3% of proteins encoded in bacterial genomes.” have been added in this revised manuscript (p. 3) to address this.

b. 60% of human drug targets are membrane proteins (again, plasma membrane proteins), but the MS is discussing bacterial proteins—drug targets for bacterial proteins are generally the ribosome or the peptidoglycan, not membrane proteins.

This sentence has been removed to avoid confusion.

6) I did not know that there was a strain of C. jejuni called "sheep abortion!" Thank you for this bit of color.

We really appreciate this reviewer’s comment. Yes, we are quite proud of discovering this strain.


Reviewer #3: This manuscript from Z. Zhang et al demonstrates the feasibility of determining high resolution structures of outer membrane proteins from cellular extracts from Campylobacter jejuni using the Gradient Enrichment of Native Targets from Lipid Environments (GENTLE) and build-and-retrieve (BaR) approach . The impact here lies within the need for high resolution structures of membrane proteins coupled with the context that C. jejuni exhibits a high-level toxin in antibiotic resistance. In addition, C. jejuni may use a distinct cell invasion mechanism with porins implicated in colonization.

The build and retrieve system enable structure determination directly from crude samples, bypassing the need for overexpression and column purification, which can destabilize proteins during lengthy purifications. Instead, sucrose cushion centrifugation is used, yielding high-resolution structures reported here; PorA/MOMP, OMP50/CjTK porins and CjSLP .

This is a strong paper that makes dual contributions to the field. The corrections are minor and are noted below

We very much appreciate this reviewer’s comment that “This is a strong paper that makes dual contributions to the field.”

Statements that need to be backed up by references :
1. Interestingly, the pore formed by each PorA protomer is negatively charged in nature (Fig 1E), suggesting that PorA may favor the shuttling of positively charged substrates.
Add citation(s) supporting the charge-based substrate selectivity claim.

A statement with a citation “This result is indeed in good agreement with the observation that PorA is capable of transporting small positively charged poly-arginine peptides across the membrane (Dhanaskekar et al. 2017. Biochem Biophys Rep 11:79-83)” has been added in this revised manuscript (p. 10) to support our observation.

2. Our cryo-EM structure of PorA-I depicts that L4 is a surface exposed loop likely responsible for direct interaction with the host.
Add citation(s) supporting the role of L4 in host interaction.

A couple of statements “In fact, of the 18 mutations observed in PorA of the hypervirulent SA strain, nine of these substitutions were found within the flexible L4. Subsequent experiments have also verified that only mutations in L4 were needed to cause abortion in guinea pig models (Wu et al. 2016. PNAS 113:10690-10695).” have been added on p. 11 of this revised manuscript to support our observation.

3.Therefore, these flexible L loops may be critical for governing the opening and closing of the OMP50 pore.
Add citation(s) supporting L-loop gating function.

This sentence has been removed. However, a new statement and a new reference “. Due to the neutrality of the inner pore combined with the arrangement of the four tyrosines (Y147, Y204, Y360 and Y399) lining the internal wall, one would expect OMP50 to be more favorable in shuttling neutral and/or positively charged ligands across the OM. Indeed, it has been documented that Omp50 is capable of forming a cation-selective pore with a major conductance of 50-60 ps (Bolla et al. 2000. Biochem J 352:637-643)” have been added on p. 14 of the revised manuscript to strengthen our finding.

4. Based on the structural information, the Cj0034c nonamer is likely to assemble as an IM channel.
Statement is logical but requires supporting examples or precedents from the literature.

We found a just published paper reporting an unusual mycobacterial outer membrane protein MctB (Siroy et al. 2026. J Biol Chem 302:111037), which utilizes an alpha helix to span the outer mycomembrane (similar to what we have seen in the case of Cj0034c). It appears that the N-terminal signaling peptide of MctB has dual functions, where it guides the expressed protein to the outer mycomembrane as well as creates an α-helical TM domain spanning the OM of the mycobacterium (Siroy et al. 2026. J Biol Chem 302:111037). Therefore, it is very likely that the signaling peptide of Cj0034c may also hold a dual-purpose to lead the protein to target the OM as well as participate in bridging the OM with an all α-helical TM domain. Our cryo-EM structure of Cj0034c may also exemplify the first structure of a Gram-negative OMP featuring with an α-helical TM domain.

A new paragraph “Based on the structural information, Cj0034c may represent an unusual OMP possessing all α-helical secondary structural elements in the TM domain. As all reported OMP structures of Gram-negative bacteria have the distinct signature of spanning the OM with β-stranded secondary elements, our structure of the Cj0034c nonamer may also exemplify the first structure of a Gram-negative OMP featuring with an α-helical TM domain. Recently, the crystal structure of an atypical mycobacterial OMP MctB (Rv1698) has been reported (Siroy et al. 2026. J Biol Chem 302:111037). The structure of this mycobacterial membrane protein also shows an α-helical membrane-spanning region across the outer mycomembrane, where the N-terminal signaling peptide has dual functions. It guides the expressed protein to the outer mycomembrane as well as creates an α-helical TM domain (Siroy et al. 2026. J Biol Chem 302:111037). It appears that the N-terminal sequence of Cj0034c may also hold a dual-purpose. It leads the protein to target the OM as well as participates in bridging the OM with an all α-helical TM domain, similar to that found in MctB.” has added on p. 18 of this revised manuscript to address this.

5. However, there is a possibility that this motif, located at the OM beta barrel, may
facilitate the binding of substrates that contain a phosphate group.
Expand on this point. Consider performing docking studies to identify or support the putative binding site.

We actually do not think this is the Walker motif, but it was indicated in a published paper (Corcionivoschi et al. 2012. Cell Host Microbe 12:47-59). In this revised manuscript, we have removed this paragraph.
