# Supplementary material for: Structural insights into the outer membrane proteins PorA, OMP50 and Cj0034c from native Campylobacter jejuni membranes
Source: PLoS Biol. 2026 Aug 28;24(8):e3003961. doi: 10.1371/journal.pbio.3003961 (PMC13759207; doi:10.1371/journal.pbio.3003961)
Supplement: Supplementary file 4 [file pbio.3003961.s016.docx]

There are three remaining concerns that need to be addressed.

1. Clarification of the GENTLE Method
Please ensure the manuscript clearly details previous descriptions and applications of the GENTLE method to provide sufficient context for the reader.

A new figure showing the GENTLE methodology has been added to this revised manuscript. In addition, we have included more details in the “Method” section to provide more information of this methodology to the reader.

2. Structural Ambiguity of OMP50-II
The addition of the OMP50-II structure is appreciated. However, the text refers to the protein as a “truncated OMP50-I,” suggesting that it is a proteolytically cleaved form of Omp50-I. When describing the structure, it is currently unclear whether the unobserved region is physically absent from the protein or if it is simply disordered. Please address this ambiguity. Performing immunoblots of tagged proteins or conducting MS analysis of cell extracts/purified components from the initial steps of the GENTLE isolation would be appropriate ways to resolve this.

The OMP50-II structure presented is not due to proteolytic cleavage of the protein, but rather it is because of the flexibility of this domain. New statements “Like PorA, this class of particles can be divided into two subsets with similar structural features, representing two distinct conformations of this OMP” (on p. 12), and “However, the cryo-EM density corresponding to the N-terminal end of the periplasmic α1-helical bundle of the structure cannot be clearly seen, indicating that the conformation of this helical bundle extending into the periplasmic space may be quite flexible in nature” (on p. 15) to clarify this. We can also remove the structure of OMP50-II (it was not presented in the original manuscript) if it is still confusing.

3. Localization of Cj0034c
The manuscript now proposes that Cj0034c is an unusual outer membrane (OM) protein, which is a pivot from the initial conclusion that it is an inner membrane (IM) protein based on its alpha-helical transmembrane (TM) region. While a rigorous experimental analysis to determine the exact location of Cj0034c may be beyond the scope of this current study, it is essential to acknowledge that the localization remains uncertain in the absence of direct experimental validation.

It is acceptable to speculate that Cj0034c is an unusual OM protein, provided it is explicitly noted that further experiments are required to validate this hypothesis. However, the current draft presents Cj0034c as an OM protein throughout the text (including the Abstract) as a certainty. For example, the Discussion states:

"Surprisingly, our cryo-EM structure indicates that full-length Cj0034c is not a periplasmic protein. Instead, it is a very unusual OMP consisting of a single TM helix and a large periplasmic domain, where the fully assembled oligomer contains nine Cj0034c protomers that form a nonameric channel spanning the OM and part of the periplasmic space of C. jejuni."

Because this study does not experimentally address the location of Cj0034c, the text must be revised carefully. Please ensure that statements formulated as hypotheses are clearly distinguished from conclusions based on direct experimental evidence.

The sections for Cj0034c have been extensively rewritten in this revised version of the manuscript.

In “Abstract”, the statements have been modified to “Our studies also led to the first structure of the full-length Cj0034c protein … However, whether Cj0034c spans the outer membrane (OM) or inner membrane (IM) of *C. jejuni* must await further experimental studies.”

In “Cj0034c/CjSLP”, a paragraph has been modified to “In Gram-negative bacteria, based upon all current reported structures, the IMP and OMP classes are distinct from each other. IMPs use α-helical structural features and OMPs utilize β-stranded secondary elements to constitute their TM domains. According to this structural information, the full-length Cj0034c membrane protein likely forms a typical IMP with a single α-helical TM from each protomer to span the IM. However, recently the crystal structure of an atypical mycobacterial OMP MctB (Rv1698) has been reported … Based on the structural information of MctB, it appears that Cj0034c could represent an unusual *G. negative* OMP possessing all α-helical secondary structural elements in the TM domain. If this is the case, then the N-terminal sequence of Cj0034c may also hold a dual-purpose. It could lead the protein to target the OM as well as participate in bridging the OM with an all α-helical TM domain, similar to that found in MctB. Therefore, the structure of the Cj0034c nonamer may exemplify the first structure of a Gram-negative OMP featuring with an α-helical TM domain. Further studies are necessary to determine the spatiotemporal nature of this membrane protein as well as its complete physiological functions.”

In “Discussion”, a paragraph has been modified to “Surprisingly, our cryo-EM structure indicates that full-length Cj0034c is not a periplasmic protein. Instead, it is a membrane protein containing nine Cj0034c protomers that form a nonameric channel, which spans the lipid bilayer and part of the periplasmic space of *C. jejuni*. Each protomer of Cj0034c constitutes a single TM α-helix and a large periplasmic domain. It is not entirely clear if full-length Cj0034c spans the IM or OM to function. Whether Cj0034c is an IMP or OMP must await further experimental confirmation. Based on the structural information, Cj0034c likely forms a typical membrane protein that spans the IM of the bacterium. If Cj0034c indeed traverses across the OM instead, then this protein would represent the first known Gram-negative OMP with its N-terminal amino acids forming both the signaling peptide and the α-helical structural elements that allow it to span the OM rather than residues that would be cleaved off after maturation as proposed.”

All changes are colored blue in the “Revised Article with Changes Highlighted”.
