# Supplementary material for: Structural insights into the outer membrane proteins PorA, OMP50 and Cj0034c from native Campylobacter jejuni membranes
Source: PLoS Biol. 2026 Aug 28;24(8):e3003961. doi: 10.1371/journal.pbio.3003961 (PMC13759207; doi:10.1371/journal.pbio.3003961)
Supplement: Supplementary file 5 [file pbio.3003961.s017.docx]

**Response to the Editor:**

(A) Could you please clarify whether you have previously published structures determined using the GENTLE method, or whether this the first publication that describes the method. If the former, could you please provide the reference? We ask because in the cover letter I note that it states the following:

“We used a methodology, termed GENTLE, that we have previously developed, to elucidate structures of C. jejuni outer membrane proteins from crude native membranes without the need to overexpress and purify them, even if the natural abundance of these protein targets is modest.”

No, we have not published the GENTLE methodology. This is our first manuscript, which documents the GENTLE methodology for determining cryo-EM structures of membrane proteins from crude native membranes. We are very sorry that the cover letter creates this confusion.

(B) We note a previous cryo-EM related method has been published with the name GENTLE (Gentle and fast all-atom model refinement to cryo-EM densities). This is a maximum-likelihood molecular dynamics refinement method developed in the following publication:

Blau, C., Yvonnesdotter, L., & Lindahl, E. (2023). Gentle and fast all-atom model refinement to cryo-EM densities via a maximum likelihood approach. PLOS Computational Biology, 19(8), e1011255. https://doi.org/10.1371/journal.pcbi.1011255).

While the Gentle approach of Blau et al does not use GENTLE as an acronym, we wonder if this could lead to confusion with two-different "gentle methods" being used for cryoEM structure determination? If you agree, then we would perhaps suggest that the method is renamed.

We would like to keep the name GENTLE if possible. It took us a long time to identify this name to represent our methodology. There is a deeper reason behind the name GENTLE too. This name can highlight the fact that our methodology is gentle, which is capable of retaining and keeping the native structures of membrane proteins and their corresponding complexes completely intact. Therefore, we would like to keep the name GENTLE.

(C) After discussions within the editorial team, we think that the ‘Short Report’ article type would be the best fit for your submission given that the manuscript lacks further functional or mechanistic follow-up. We also note that the manuscript contains 3 main data figures, so is a natural fit in this article type since they have a maximum of 4 main figures. Given our previous discussions about this, please do let me know if you would like to discuss this or if you have any further questions or concerns.

Yes, this is totally fine. A ‘Short Report’ article is a good idea.

(D) We routinely suggest changes to titles to ensure maximum accessibility for a broad, non-specialist readership. In this case, we would suggest the following edit to the title for flow and additional information, as follows. Please ensure you change both the manuscript file and the online submission system, as they need to match for final acceptance:

“Structural insights into the outer membrane proteins PorA, OMP50 and Cj0034c from native Campylobacter jejuni membranes”

Yes, we agree. This title is good.

(E) Thank you for depositing the structural data in the PDB and EMDB database. However, we note that the structures are currently on hold for release. We ask that you please make the structures publicly available at this stage before publication.

Yes, we have already requested to release the structures to the public. All PDBs and EMDBs have been scheduled to be release to the public by August 12, 2026.

(F) In addition, please make the mass spectrometry data deposited in the ProteomeXchange publicly available (PXD072772).

Yes, we have already requested to release the mass spectrometry data to the public. ProteomeXchange will release the raw mass spectrometry data within two days as soon as they receive the DOI number of this manuscript.

(G) Please also ensure that each of the relevant figure legends in your manuscript include information on *WHERE THE UNDERLYING DATA CAN BE FOUND*, and ensure your supplemental data file/s has a legend.

Yes, this information has been added.

(H) Per journal policy, if you have generated any custom code during the course of this investigation, please make it available without restrictions. Please ensure that the code is sufficiently well documented and reusable, and that your Data Statement in the Editorial Manager submission system accurately describes where your code can be found. More information on our Code Policy, what and how to share can be found here: https://journals.plos.org/plosbiology/s/code-availability

Yes, all atomic coordinates and cryo-EM maps are made available (with accession codes) to the public without restrictions. The mass spectrometry proteomics data are also made available (with an identifier) to the public without restrictions.

Please note that we cannot accept sole deposition of code in GitHub, as this could be changed after publication. However, you can archive this version of your publicly available GitHub code to Zenodo. Once you do this, it will generate a DOI number, which you will need to provide in the Data Accessibility Statement (you are welcome to also provide the GitHub access information). See the process for doing this here: https://docs.github.com/en/repositories/archiving-a-github-repository/referencing-and-citing-content


(I) Please ensure that your Data Statement in the submission system accurately describes where your data can be found and is in final format, as it will be published as written there.

Yes, the data statement accurately describes where the data can be found and what the final format is.

----------------------------------------------------------------

COMMENTS FROM THE ACADEMIC EDITOR

I do not think the authors have answered why they think that the poor density for the periplasmic region of OMP50-II (relative to OMP50-I) is indicative of flexibility in the N-terminal end rather than the region being altogether missing (previous point #3). Can they show some density for this region, even if it is poorly resolved? If not then why are they ruling out proteolysis?

A new supplementary figure S7 showing cryo-EM densities of OMP50-II at three different contours, is included in this revised version of the manuscript. By lowering the contour level of the map, we indeed can observe densities corresponding to the α1-helical bundle. This observation suggests that it is more likely that the N-terminal residues of this protein are quite flexible.
