# Supplementary material for: Structural insights into the outer membrane proteins PorA, OMP50 and Cj0034c from native Campylobacter jejuni membranes
Source: PLoS Biol. 2026 Aug 28;24(8):e3003961. doi: 10.1371/journal.pbio.3003961 (PMC13759207; doi:10.1371/journal.pbio.3003961)
Supplement: Peer review history

# Editor report: Decision Letter 0

**DOI:** 10.1371/journal.pbio.3003961.r001

**Authors:** Richard Hodge

18 Feb 2026

Dear Ed,

Thank you for submitting your manuscript entitled "Targeting outer membrane proteins (OMPs) directly from crude Campylobacter jejuni membranes for structural determination" for consideration as a Research Article by PLOS Biology.

Your manuscript has now been evaluated by the PLOS Biology editorial staff, as well as by an academic editor with relevant expertise, and I am writing to let you know that we would like to send your submission out for external peer review.

IMPORTANT: After discussions within the editorial team, we think that your manuscript would be a better fit as a 'Short Report' at the journal (https://journals.plos.org/plosbiology/s/what-we-publish#loc-short-reports). Upon resubmission (guidelines below), I would be grateful if you could please tick 'Short Report' as the article type in the dropdown menu in the online submission form.

Before we can send your manuscript to reviewers, we need you to complete your submission by providing the metadata that is required for full assessment. To this end, please login to Editorial Manager where you will find the paper in the 'Submissions Needing Revisions' folder on your homepage. Please click 'Revise Submission' from the Action Links and complete all additional questions in the submission questionnaire.

Once your full submission is complete, your paper will undergo a series of checks in preparation for peer review. After your manuscript has passed the checks it will be sent out for review. To provide the metadata for your submission, please Login to Editorial Manager (https://www.editorialmanager.com/pbiology) within two working days, i.e. by Feb 20 2026 11:59PM.

If your manuscript has been previously peer-reviewed at another journal, PLOS Biology is willing to work with those reviews in order to avoid re-starting the process. Submission of the previous reviews is entirely optional and our ability to use them effectively will depend on the willingness of the previous journal to confirm the content of the reports and share the reviewer identities. Please note that we reserve the right to invite additional reviewers if we consider that additional/independent reviewers are needed, although we aim to avoid this as far as possible. In our experience, working with previous reviews does save time.

If you would like us to consider previous reviewer reports, please edit your cover letter to let us know and include the name of the journal where the work was previously considered and the manuscript ID it was given. In addition, please upload a response to the reviews as a 'Prior Peer Review' file type, which should include the reports in full and a point-by-point reply detailing how you have or plan to address the reviewers' concerns.

During the process of completing your manuscript submission, you will be invited to opt-in to posting your pre-review manuscript as a bioRxiv preprint. Visit http://journals.plos.org/plosbiology/s/preprints for full details. If you consent to posting your current manuscript as a preprint, please upload a single Preprint PDF.

Feel free to email us at plosbiology@plos.org if you have any queries relating to your submission.

Kind regards,

Richard

Richard Hodge, PhD

Senior Editor, PLOS Biology

rhodge@plos.org

PLOS

Empowering researchers to transform science

Carlyle House, Carlyle Road, Cambridge, CB4 3DN, United Kingdom

California (U.S.) corporation #C2354500, based in San Francisco

# Peer review: Decision Letter 1

**DOI:** 10.1371/journal.pbio.3003961.r002

**Authors:** Richard Hodge

27 Mar 2026

Dear Ed,

Thank you for your patience while your manuscript "Targeting outer membrane proteins (OMPs) directly from crude Campylobacter jejuni membranes for structural determination" was peer-reviewed at PLOS Biology. Please accept my apologies for the delays that you have experienced during the peer review process. Your manuscript has now been evaluated by the PLOS Biology editors, an Academic Editor with relevant expertise, and by three independent reviewers.

In light of the reviews, which you will find at the end of this email, we would like to invite you to revise the work to thoroughly address the reviewers' reports.

As you will see, the reviewers are generally positive about your manuscript and think the structures are interesting and well done. However, they raise several concerns that should be addressed in a revised version. Reviewer #1 notes the description and reporting of the GENTLE methodology should be expanded in the Methods section and we also ask that it is made clearer in the text that the methods were described in a previous publication. In addition, the reviewers raise overlapping concerns with the strength of the data to support the claims that Cj0034c is localized to the inner membrane. After discussions with the academic editor, we think that additional experimental work should be provided to provide a rigorous demonstration that Cj0034c is an inner membrane channel.

Given the extent of revision needed, we cannot make a decision about publication until we have seen the revised manuscript and your response to the reviewers' comments. Your revised manuscript is likely to be sent for further evaluation by all or a subset of the reviewers.

In addition to these revisions, you will need to complete some formatting changes, which you will receive in a follow up email. A member of our team will be in touch with a set of requests shortly.

We expect to receive your revised manuscript within 3 months. Please email us (plosbiology@plos.org) if you have any questions or concerns, or would like to request an extension.

At this stage, your manuscript remains formally under active consideration at our journal; please notify us by email if you do not intend to submit a revision so that we may withdraw it.

**IMPORTANT - SUBMITTING YOUR REVISION**

Your revisions should address the specific points made by each reviewer. Please submit the following files along with your revised manuscript:

1. A 'Response to Reviewers' file - this should detail your responses to the editorial requests, present a point-by-point response to all of the reviewers' comments, and indicate the changes made to the manuscript.

*NOTE: In your point-by-point response to the reviewers, please provide the full context of each review. Do not selectively quote paragraphs or sentences to reply to. The entire set of reviewer comments should be present in full and each specific point should be responded to individually, point by point.

You should also cite any additional relevant literature that has been published since the original submission and mention any additional citations in your response.

2. In addition to a clean copy of the manuscript, please also upload a 'track-changes' version of your manuscript that specifies the edits made. This should be uploaded as a "Revised Article with Changes Highlighted" file type.

*Re-submission Checklist*

When you are ready to resubmit your revised manuscript, please refer to this re-submission checklist: https://plos.io/Biology_Checklist

To submit a revised version of your manuscript, please go to https://www.editorialmanager.com/pbiology/ and log in as an Author. Click the link labelled 'Submissions Needing Revision' where you will find your submission record.

Please make sure to read the following important policies and guidelines while preparing your revision:

*Published Peer Review*

Please note while forming your response, if your article is accepted, you may have the opportunity to make the peer review history publicly available. The record will include editor decision letters (with reviews) and your responses to reviewer comments. If eligible, we will contact you to opt in or out. Please see here for more details:

https://blogs.plos.org/plos/2019/05/plos-journals-now-open-for-published-peer-review/

*PLOS Data Policy*

Please note that as a condition of publication PLOS' data policy (http://journals.plos.org/plosbiology/s/data-availability) requires that you make available all data used to draw the conclusions arrived at in your manuscript. If you have not already done so, you must include any data used in your manuscript either in appropriate repositories, within the body of the manuscript, or as supporting information (N.B. this includes any numerical values that were used to generate graphs, histograms etc.). For an example see here: http://www.plosbiology.org/article/info%3Adoi%2F10.1371%2Fjournal.pbio.1001908#s5

*Blot and Gel Data Policy*

We require the original, uncropped and minimally adjusted images supporting all blot and gel results reported in an article's figures or Supporting Information files. We will require these files before a manuscript can be accepted so please prepare them now, if you have not already uploaded them. Please carefully read our guidelines for how to prepare and upload this data: https://journals.plos.org/plosbiology/s/figures#loc-blot-and-gel-reporting-requirements

*Protocols deposition*

To enhance the reproducibility of your results, we recommend that if applicable you deposit your laboratory protocols in protocols.io, where a protocol can be assigned its own identifier (DOI) such that it can be cited independently in the future. Additionally, PLOS ONE offers an option for publishing peer-reviewed Lab Protocol articles, which describe protocols hosted on protocols.io. Read more information on sharing protocols at https://plos.org/protocols?utm_medium=editorial-email&utm_source=authorletters&utm_campaign=protocols

Thank you again for your submission to our journal. We hope that our editorial process has been constructive thus far, and we welcome your feedback at any time. Please don't hesitate to contact us if you have any questions or comments.

Best regards,

Richard

Richard Hodge, PhD

Senior Editor, PLOS Biology

rhodge@plos.org

------------------------------------

REVIEWS:

Reviewer #1 (Bert van den Berg, identifies himself): The manuscript by Yu and colleagues describes an application of the BaR methodology, where total membranes are isolated followed by cryo-EM structure determination of the most abundant/stable complexes. In this case the target organism is the Gram-negative pathogen Campylobacter jejuni, and the membranes are isolated via mild detergent (LMNG) extraction combined with sucrose gradient centrifugation. The authors are able to solve cryo-EM structures of three membrane proteins: PorA (or MOMP), Omp50 and Cj0034c. The first two are outer membrane proteins (OMPs) and the last one an unexpected inner membrane protein (IMP) of unknown function. The structures of PorA generate some extra, potentially important information that add on insights garnered from previous structures determined by X-ray crystallography, i.e. the role of calcium in the conformation of the functionally important loop L4. No previous structures were determined for Omp50, and the current structure identifies a trimer with a periplasmic coiled coil as well as narrow channels in each trimer that are lined by some tyrosine residues. Finally, the Cj0034c structure presents a major surprise since the structure suggests (but see comments below) that it forms a nonameric IM channel with a large periplasmic domain, which contrast with previous notions based on crystallography (soluble periplasmic protein).

Overall, this is an interesting application of the BaR method that was developed and previously published by the authors. The structures are beautiful and skillfully done, and the manuscript is overall reasonably well-written, but not very reviewer-friendly (see comment 1). My main issue with the paper, combined with the fact that PLOS Biology publication is sought, is that it represents a bit of "stamp collecting", where the methodology can be applied in a fairly straightforward way to any organism of interest to solve MP structures. There are no hypothesis being tested regarding any of the interesting biology of Campylobacter jejuni (e.g. the role of Omp50 in Tyr phosphorylation), despite the wealth of information that is provided by the structures. Besides this, I have some comments for consideration by the authors:

1. There are no subheadings in the text. Also, line numbers are missing. Finally, it would be appreciated if (at least) the main figures with their legends are inserted within the text.

2. On page 10, the authors say that the Omp50 architecture is "unique". In which way? 16-stranded beta-barrel trimers are every common, tyrosines also occur in large numbers in OMPs, and a helical coiled-coil was also recently reported in an OMP (PMID: 37932269). Please tone this down. There are also other instances where results and implications are overstated. Like I said above, it is a pity that the authors did not attempt to study more of the biology of this interesting protein now that they solved the structure. On page 11 it is implied that 27 tyrosine residues are a consequence of the function of the protein, without evidence being provided. Tyrosines are generally very abundant in OMPs.

3. The statement on page 12 at the end of the first paragraph is complete speculation. The same goes for the last sentence of the second paragraph. Also, the apparent fact that loop L4 forms part of the "valve" (is that the correct term?) of the neighbouring pore is not well shown.

4. Top page 13: is this a real Walker motif? Please show its location. Note also that the presence of ATP in any bacterial periplasmic space would not be expected.

5. The most puzzling of the new structures is that of Cj0034c. If it is IM localised/periplasmic, how could it be involved in adhesion and how could it be an attractive vaccine candidate? Or is this all based on faulty assumptions in previous studies (eg refs 42 and 43)? But if this protein is an IMP, the fairly large channel would present a serious breach of the IM barrier. It is very unlikely to "directly connect the periplasm with the cytoplasm", as stated on page 18. Is it possible that we are dealing with a highly unusual hydrophobic TM segment that is somehow inserted into the OM? The presence of an MFS type transporter within the operon (how do we know this?) would also suggest that this couples somehow to Cj0034c to form a channel across the periplasmic space (inserted into the OM). Has the gene been annotated correctly (is there an alternative start codon with a signal sequence). The open channel is just extremely unlikely to insert into the IM. The authors do not critically analyse the important implications of the structure and the previous literature. Finally, the structure by itself doesn't allow one to say conclusively whether a protein is IM or OM-localised.

6. Page 17: rephrase "all 27 tyrosines, which are important for.......". What is then this network of tyrosines that the structure is purported to show, given that OMPs contain many Tyr residues?

7. The notion that GENTLE will be powerful for determination of fragile envelope-spanning complexes is not supported by the data. OMPs are known to be stable and the structure of Cj0034c also suggests it is very stable. If the others want to test this notion they should overexpress an efflux pump and see if they isolate a complex. This also brings me to the proteomics table: why are there no IMP complexes present within the data (or are there)? Is the method not as gentle as we are led to believe?

8. Methods: the section describing GENTLE is poor. It is important to include volumes of buffer, amount of membranes etc in order to ensure this can be repeated exactly. Also centrifuge used, tubes etc. After centrifugation, how is the membrane pellet dissolved exactly?

9. Proteomics: "10 ug of membrane sample". Based on BCA (proteins) or weight?

10. The main figures are quite fuzzy for some reason in the provided PDF.

Reviewer #2: This MS describes the use of an exciting method for identification and solving of cryo-EM structures from bacterial cells without specific overexpression. The authors use the method to solve 3-4 structures of membrane proteins connected to the virulence of C. jejuni. This manuscript is chockful of insights about the three/four structurally characterized proteins.

Although I see that this MS has already gone through one round of revision, I have not seen the explanation of changes and did not review the initial submission.

A few questions and requests for improvement:

1)An important insight of this manuscript is that the GENTLE methodology allowed for identification and solving of cryo-EM structures of OMPs from native C. jejuni membranes without overexpression. And that this method is not restricted to proteins of high abundance. This point would be better illustrated if a table with relative abundances of the proteins solved as well as maybe some abundances of higher and lower abundance proteins to better understand the un-restrictedness and/or limitations of this method.

2)There is quite a bit of repetition in the section about the characterization of CjSLP. It reads like that section was written twice but one of the versions was never removed. Could these two sections be merged more effectively?

3)What does the SLP of CjSLP stand for?

4)How is it clear that CjSLP is an inner membrane protein and not an unusual helical protein in the outer membrane?

5)The first paragraph of the introduction is misleading in two ways:

a.Yes, 20-30% of proteins are membrane proteins but that's all inner membrane/plasma membrane proteins. The outer membrane proteins that this MS describes are 1 -2 % of the proteins of Campylobacter.

b.60% of human drug targets are membrane proteins (again, plasma membrane proteins), but the MS is discussing bacterial proteins—drug targets for bacterial proteins are generally the ribosome or the peptidoglycan, not membrane proteins.

6)I did not know that there was a strain of C. jejuni called "sheep abortion!" Thank you for this bit of color.

Reviewer #3: This manuscript from Z. Zhang et al demonstrates the feasibility of determining high resolution structures of outer membrane proteins from cellular extracts from Campylobacter jejuni

using the Gradient Enrichment of Native Targets from Lipid Environments (GENTLE) and build-and-retrieve (BaR) approach . The impact here lies within the need for high resolution structures of membrane proteins coupled with the context that C. jejuni exhibits a high-level toxin in antibiotic resistance. In addition, C. jejuni may use a distinct cell invasion mechanism with porins implicated in colonization.

The build and retrieve system enable structure determination directly from crude samples, bypassing the need for overexpression and column purification, which can destabilize proteins during lengthy purifications. Instead, sucrose cushion centrifugation is used, yielding high-resolution structures reported here; PorA/MOMP, OMP50/CjTK porins and CjSLP .

This is a strong paper that makes dual contributions to the field. The corrections are minor and are noted below

Statements that need to be backed up by references :

1. Interestingly, the pore formed by each PorA protomer is negatively charged in nature (Fig 1E), suggesting that PorA may favor the shuttling of positively charged substrates.

Add citation(s) supporting the charge-based substrate selectivity claim.

2. Our cryo-EM structure of PorA-I depicts that L4 is a surface exposed loop likely responsible for direct interaction with the host.

Add citation(s) supporting the role of L4 in host interaction.

3.Therefore, these flexible L loops may be critical for governing the opening and closing of the OMP50 pore.

Add citation(s) supporting L-loop gating function.

4. Based on the structural information, the Cj0034c nonamer is likely to assemble as an IM channel.

Statement is logical but requires supporting examples or precedents from the literature.

5. However, there is a possibility that this motif, located at the OM beta barrel, may

facilitate the binding of substrates that contain a phosphate group.

Expand on this point. Consider performing docking studies to identify or support the putative binding site.

# Author comment: Author response to Decision Letter 2

**DOI:** 10.1371/journal.pbio.3003961.r003

30 Apr 2026

Attachment Submitted filename: Point-by-point response to reviewers PLoS Biol OMP.docx

# Editor report: Decision Letter 2

**DOI:** 10.1371/journal.pbio.3003961.r004

**Authors:** Richard Hodge

12 May 2026

Dear Dr Yu,

Thank you for your patience while we considered your revised manuscript "Targeting outer membrane proteins (OMPs) directly from crude Campylobacter jejuni membranes for structural determination using the "Gradient Enrichment of Native Targets from Lipid Enrichments" (GENTLE) methodology" for publication as a Short Reports at PLOS Biology. Your revised study has been evaluated by the PLOS Biology editors and the Academic Editor.

After discussion with the academic editor, whose comments you will find at the end of this email, we would like to invite you to revise the work to thoroughly address the three remaining comments.

Given the extent of revision needed, we cannot make a decision about publication until we have seen the revised manuscript. Your revised manuscript may be sent for further evaluation by all or a subset of the reviewers.

In addition to these revisions, you will need to complete some formatting changes, which you will receive in a follow up email. A member of our team will be in touch with a set of requests shortly.

We expect to receive your revised manuscript within 3 months. Please email us (plosbiology@plos.org) if you have any questions or concerns, or would like to request an extension.

At this stage, your manuscript remains formally under active consideration at our journal; please notify us by email if you do not intend to submit a revision so that we may withdraw it.

**IMPORTANT - SUBMITTING YOUR REVISION**

Your revisions should address the specific points made by each reviewer. Please submit the following files along with your revised manuscript:

1. A 'Response to Reviewers' file - this should detail your responses to the editorial requests, present a point-by-point response to all of the reviewers' comments, and indicate the changes made to the manuscript.

*NOTE: In your point-by-point response to the reviewers, please provide the full context of each review. Do not selectively quote paragraphs or sentences to reply to. The entire set of reviewer comments should be present in full and each specific point should be responded to individually, point by point.

You should also cite any additional relevant literature that has been published since the original submission and mention any additional citations in your response.

2. In addition to a clean copy of the manuscript, please also upload a 'track-changes' version of your manuscript that specifies the edits made. This should be uploaded as a "Revised Article with Changes Highlighted" file type.

*Re-submission Checklist*

When you are ready to resubmit your revised manuscript, please refer to this re-submission checklist: https://plos.io/Biology_Checklist

To submit a revised version of your manuscript, please go to https://www.editorialmanager.com/pbiology/ and log in as an Author. Click the link labelled 'Submissions Needing Revision' where you will find your submission record.

Please make sure to read the following important policies and guidelines while preparing your revision:

*Published Peer Review*

Please note while forming your response, if your article is accepted, you may have the opportunity to make the peer review history publicly available. The record will include editor decision letters (with reviews) and your responses to reviewer comments. If eligible, we will contact you to opt in or out. Please see here for more details:

https://blogs.plos.org/plos/2019/05/plos-journals-now-open-for-published-peer-review/

*PLOS Data Policy*

Please note that as a condition of publication PLOS' data policy (http://journals.plos.org/plosbiology/s/data-availability) requires that you make available all data used to draw the conclusions arrived at in your manuscript. If you have not already done so, you must include any data used in your manuscript either in appropriate repositories, within the body of the manuscript, or as supporting information (N.B. this includes any numerical values that were used to generate graphs, histograms etc.). For an example see here: http://www.plosbiology.org/article/info%3Adoi%2F10.1371%2Fjournal.pbio.1001908#s5

*Blot and Gel Data Policy*

We require the original, uncropped and minimally adjusted images supporting all blot and gel results reported in an article's figures or Supporting Information files. We will require these files before a manuscript can be accepted so please prepare them now, if you have not already uploaded them. Please carefully read our guidelines for how to prepare and upload this data: https://journals.plos.org/plosbiology/s/figures#loc-blot-and-gel-reporting-requirements

*Protocols deposition*

To enhance the reproducibility of your results, we recommend that if applicable you deposit your laboratory protocols in protocols.io, where a protocol can be assigned its own identifier (DOI) such that it can be cited independently in the future. Additionally, PLOS ONE offers an option for publishing peer-reviewed Lab Protocol articles, which describe protocols hosted on protocols.io. Read more information on sharing protocols at https://plos.org/protocols?utm_medium=editorial-email&utm_source=authorletters&utm_campaign=protocols

Thank you again for your submission to our journal. We hope that our editorial process has been constructive thus far, and we welcome your feedback at any time. Please don't hesitate to contact us if you have any questions or comments.

Sincerely,

Christian

Christian Schnell, PhD

Senior Editor

PLOS Biology

cschnell@plos.org

on behalf of

Richard Hodge, PhD

Senior Editor

PLOS Biology

rhodge@plos.org

------------------------------------

There are three remaining concerns that need to be addressed.

1. Clarification of the GENTLE Method

Please ensure the manuscript clearly details previous descriptions and applications of the GENTLE method to provide sufficient context for the reader.

2. Structural Ambiguity of OMP50-II

The addition of the OMP50-II structure is appreciated. However, the text refers to the protein as a “truncated OMP50-I,” suggesting that it is a proteolytically cleaved form of Omp50-I. When describing the structure, it is currently unclear whether the unobserved region is physically absent from the protein or if it is simply disordered. Please address this ambiguity. Performing immunoblots of tagged proteins or conducting MS analysis of cell extracts/purified components from the initial steps of the GENTLE isolation would be appropriate ways to resolve this.

3. Localization of Cj0034c

The manuscript now proposes that Cj0034c is an unusual outer membrane (OM) protein, which is a pivot from the initial conclusion that it is an inner membrane (IM) protein based on its alpha-helical transmembrane (TM) region. While a rigorous experimental analysis to determine the exact location of Cj0034c may be beyond the scope of this current study, it is essential to acknowledge that the localization remains uncertain in the absence of direct experimental validation.

It is acceptable to speculate that Cj0034c is an unusual OM protein, provided it is explicitly noted that further experiments are required to validate this hypothesis. However, the current draft presents Cj0034c as an OM protein throughout the text (including the Abstract) as a certainty. For example, the Discussion states:

"Surprisingly, our cryo-EM structure indicates that full-length Cj0034c is not a periplasmic protein. Instead, it is a very unusual OMP consisting of a single TM helix and a large periplasmic domain, where the fully assembled oligomer contains nine Cj0034c protomers that form a nonameric channel spanning the OM and part of the periplasmic space of C. jejuni."

Because this study does not experimentally address the location of Cj0034c, the text must be revised carefully. Please ensure that statements formulated as hypotheses are clearly distinguished from conclusions based on direct experimental evidence.

# Author comment: Author response to Decision Letter 3

**DOI:** 10.1371/journal.pbio.3003961.r005

13 Jul 2026

Attachment Submitted filename: Respond to the PLoS Biol Editor.docx

# Editor report: Decision Letter 3

**DOI:** 10.1371/journal.pbio.3003961.r006

**Authors:** Richard Hodge

26 Jul 2026

Dear Ed,

Thank you for your patience while we considered your revised manuscript "Targeting outer membrane proteins (OMPs) directly from crude Campylobacter jejuni membranes for structural determination using the "Gradient Enrichment of Native Targets from Lipid Environments" (GENTLE) methodology" for consideration as a Short Report at PLOS Biology. Please accept my sincere apologies for the delay in getting back to you as the academic editor assessed the revision and rebuttal, especially. Your revised study has now been evaluated by the PLOS Biology editors and the Academic Editor.

We are pleased to offer you the opportunity to address the remaining point from the academic editor in a revision that we anticipate should not take you very long. This comment from the academic editor is pasted underneath my signature (labelled 'Comments from the Academic Editor'). We will then assess your revised manuscript and your response to the reviewers' comments with our Academic Editor aiming to avoid further rounds of peer-review.

In addition, please make sure to address the following editorial and data-related requests that I have provided below (A-I):

(A) Could you please clarify whether you have previously published structures determined using the GENTLE method, or whether this the first publication that describes the method. If the former, could you please provide the reference? We ask because in the cover letter I note that it states the following:

“We used a methodology, termed GENTLE, that we have previously developed, to elucidate structures of C. jejuni outer membrane proteins from crude native membranes without the need to overexpress and purify them, even if the natural abundance of these protein targets is modest.”

(B) We note a previous cryo-EM related method has been published with the name GENTLE (Gentle and fast all-atom model refinement to cryo-EM densities). This is a maximum-likelihood molecular dynamics refinement method developed in the following publication:

Blau, C., Yvonnesdotter, L., & Lindahl, E. (2023). Gentle and fast all-atom model refinement to cryo-EM densities via a maximum likelihood approach. PLOS Computational Biology, 19(8), e1011255. https://doi.org/10.1371/journal.pcbi.1011255).

While the Gentle approach of Blau et al does not use GENTLE as an acronym, we wonder if this could lead to confusion with two-different "gentle methods" being used for cryoEM structure determination? If you agree, then we would perhaps suggest that the method is renamed.

(C) After discussions within the editorial team, we think that the ‘Short Report’ article type would be the best fit for your submission given that the manuscript lacks further functional or mechanistic follow-up. We also note that the manuscript contains 3 main data figures, so is a natural fit in this article type since they have a maximum of 4 main figures. Given our previous discussions about this, please do let me know if you would like to discuss this or if you have any further questions or concerns.

(D) We routinely suggest changes to titles to ensure maximum accessibility for a broad, non-specialist readership. In this case, we would suggest the following edit to the title for flow and additional information, as follows. Please ensure you change both the manuscript file and the online submission system, as they need to match for final acceptance:

“Structural insights into the outer membrane proteins PorA, OMP50 and Cj0034c from native Campylobacter jejuni membranes”

(E) Thank you for depositing the structural data in the PDB and EMDB database. However, we note that the structures are currently on hold for release. We ask that you please make the structures publicly available at this stage before publication.

(F) In addition, please make the mass spectrometry data deposited in the ProteomeXchange publicly available (PXD072772).

(G) Please also ensure that each of the relevant figure legends in your manuscript include information on *WHERE THE UNDERLYING DATA CAN BE FOUND*, and ensure your supplemental data file/s has a legend.

(H) Per journal policy, if you have generated any custom code during the course of this investigation, please make it available without restrictions. Please ensure that the code is sufficiently well documented and reusable, and that your Data Statement in the Editorial Manager submission system accurately describes where your code can be found. More information on our Code Policy, what and how to share can be found here: https://journals.plos.org/plosbiology/s/code-availability

Please note that we cannot accept sole deposition of code in GitHub, as this could be changed after publication. However, you can archive this version of your publicly available GitHub code to Zenodo. Once you do this, it will generate a DOI number, which you will need to provide in the Data Accessibility Statement (you are welcome to also provide the GitHub access information). See the process for doing this here: https://docs.github.com/en/repositories/archiving-a-github-repository/referencing-and-citing-content

(I) Please ensure that your Data Statement in the submission system accurately describes where your data can be found and is in final format, as it will be published as written there.

----------------------------------------------------------------

As you address these items, please take this last chance to review your reference list to ensure that it is complete and correct. If you have cited papers that have been retracted, please include the rationale for doing so in the manuscript text, or remove these references and replace them with relevant current references. Any changes to the reference list should be mentioned in the cover letter that accompanies your revised manuscript.

In addition to these revisions, you may need to complete some formatting changes, which you will receive in a follow up email. A member of our team will be in touch with a set of requests shortly. If you do not receive a separate email within a few days, please assume that checks have been completed, and no additional changes are required.

We expect to receive your revised manuscript within 1 month. Please email us (plosbiology@plos.org) if you have any questions or concerns, or would like to request an extension.

At this stage, your manuscript remains formally under active consideration at our journal; please notify us by email if you do not intend to submit a revision so that we withdraw the manuscript.

**IMPORTANT - SUBMITTING YOUR REVISION**

Your revisions should address the specific points made by each reviewer. Please submit the following files along with your revised manuscript:

1. A 'Response to Reviewers' file - this should detail your responses to the editorial requests, present a point-by-point response to all of the reviewers' comments, and indicate the changes made to the manuscript.

*NOTE: In your point-by-point response to the reviewers, please provide the full context of each review. Do not selectively quote paragraphs or sentences to reply to. The entire set of reviewer comments should be present in full and each specific point should be responded to individually.

You should also cite any additional relevant literature that has been published since the original submission and mention any additional citations in your response.

2. In addition to a clean copy of the manuscript, please also upload a 'track-changes' version of your manuscript that specifies the edits made. This should be uploaded as a "Revised Article with Changes Highlighted " file type.

*Resubmission Checklist*

When you are ready to resubmit your revised manuscript, please refer to this resubmission checklist: https://plos.io/Biology_Checklist

To submit a revised version of your manuscript, please go to https://www.editorialmanager.com/pbiology/ and log in as an Author. Click the link labelled 'Submissions Needing Revision' where you will find your submission record.

Please make sure to read the following important policies and guidelines while preparing your revision:

*Published Peer Review*

Please note while forming your response, if your article is accepted, you may have the opportunity to make the peer review history publicly available. The record will include editor decision letters (with reviews) and your responses to reviewer comments. If eligible, we will contact you to opt in or out. Please see here for more details:

https://blogs.plos.org/plos/2019/05/plos-journals-now-open-for-published-peer-review/

*PLOS Data Policy*

Please note that as a condition of publication PLOS' data policy (http://journals.plos.org/plosbiology/s/data-availability) requires that you make available all data used to draw the conclusions arrived at in your manuscript. If you have not already done so, you must include any data used in your manuscript either in appropriate repositories, within the body of the manuscript, or as supporting information (N.B. this includes any numerical values that were used to generate graphs, histograms etc.). For an example see here: http://www.plosbiology.org/article/info%3Adoi%2F10.1371%2Fjournal.pbio.1001908#s5

*Blot and Gel Data Policy*

We require the original, uncropped and minimally adjusted images supporting all blot and gel results reported in an article's figures or Supporting Information files. We will require these files before a manuscript can be accepted so please prepare them now, if you have not already uploaded them. Please carefully read our guidelines for how to prepare and upload this data: https://journals.plos.org/plosbiology/s/figures#loc-blot-and-gel-reporting-requirements

*Protocols deposition*

To enhance the reproducibility of your results, we recommend that if applicable you deposit your laboratory protocols in protocols.io, where a protocol can be assigned its own identifier (DOI) such that it can be cited independently in the future. Additionally, PLOS ONE offers an option for publishing peer-reviewed Lab Protocol articles, which describe protocols hosted on protocols.io. Read more information on sharing protocols at https://plos.org/protocols?utm_medium=editorial-email&utm_source=authorletters&utm_campaign=protocols

Thank you again for your submission to our journal. We hope that our editorial process has been constructive thus far, and we welcome your feedback at any time. Please don't hesitate to contact us if you have any questions or comments.

Best regards,

Richard

Richard Hodge, PhD

Senior Editor, PLOS Biology

rhodge@plos.org

PLOS

Empowering researchers to transform science

Carlyle House, Carlyle Road, Cambridge, CB4 3DN, United Kingdom

California (U.S.) corporation #C2354500, based in San Francisco

COMMENTS FROM THE ACADEMIC EDITOR

I do not think the authors have answered why they think that the poor density for the periplasmic region of OMP50-II (relative to OMP50-I) is indicative of flexibility in the N-terminal end rather than the region being altogether missing (previous point #3). Can they show some density for this region, even if it is poorly resolved? If not then why are they ruling out proteolysis?

# Author comment: Author response to Decision Letter 4

**DOI:** 10.1371/journal.pbio.3003961.r007

5 Aug 2026

Attachment Submitted filename: Response to Reviewers.docx

# Editor report: Decision Letter 4

**DOI:** 10.1371/journal.pbio.3003961.r008

**Authors:** Richard Hodge

6 Aug 2026

Dear Ed,

On behalf of my colleagues and the Academic Editor, Ann Stock, I am pleased to say that we can accept your manuscript for publication, provided you address any remaining formatting and reporting issues. These will be detailed in an email you should receive within 2-3 business days from our colleagues in the journal operations team; no action is required from you until then. Please note that we will not be able to formally accept your manuscript and schedule it for publication until you have completed any requested changes.

Please take a minute to log into Editorial Manager at http://www.editorialmanager.com/pbiology/, click the "Update My Information" link at the top of the page, and update your user information to ensure an efficient production process.

PRESS

We frequently collaborate with press offices. If your institution or institutions have a press office, please notify them about your upcoming paper at this point, to enable them to help maximise its impact. If the press office is planning to promote your findings, we would be grateful if they could coordinate with biologypress@plos.org. If you have previously opted in to the early version process, we ask that you notify us immediately of any press plans so that we may opt out on your behalf.

We also ask that you take this opportunity to read our Embargo Policy regarding the discussion, promotion and media coverage of work that is yet to be published by PLOS. As your manuscript is not yet published, it is bound by the conditions of our Embargo Policy. Please be aware that this policy is in place both to ensure that any press coverage of your article is fully substantiated and to provide a direct link between such coverage and the published work. For full details of our Embargo Policy, please visit http://www.plos.org/about/media-inquiries/embargo-policy/.

Thank you again for choosing PLOS Biology for publication and supporting Open Access publishing. We look forward to publishing your study.

Best wishes,

Richard

Richard Hodge, PhD

Senior Editor, PLOS Biology

rhodge@plos.org

PLOS

Empowering researchers to transform science

Carlyle House, Carlyle Road, Cambridge, CB4 3DN, United Kingdom

California (U.S.) corporation #C2354500, based in San Francisco
